# Acceleration of Lactate Uptake and Utilization Contributes to Neuroprotective Action of FGF21 Involved in Naturally Aging Mice

**DOI:** 10.1111/acel.70423

**Published:** 2026-02-21

**Authors:** Keru Ji, Hongde Wei, Ruguang Wang, Yutong Wu, Chen Li, Hongchang Gao, Liangcai Zhao

**Affiliations:** ^1^ State Key Laboratory of Macromolecular Drugs and Large‐Scale Manufacturing, School of Pharmaceutical Sciences Wenzhou Medical University Wenzhou Zhejiang China; ^2^ Oujiang Laboratory (Zhejiang Lab for Regenerative Medicine, Vision and Brain Health), Institute of Metabonomics & Medical NMR Wenzhou Medical University Wenzhou China

**Keywords:** aging, ANLS, FGF21, HCA1, lactate homeostasis, MCT2

## Abstract

Brain aging is characterized by neuroinflammation and lactate metabolic changes. However, the functional role of FGF21 in the aging brain and its influence on lactate homeostasis remains unclear until now. In the study, male C57BL/6 mice were divided into 2‐month‐old (control), 20‐month (aging), and FGF21‐treated aging mice (FGF21). We also examined the MAPK signals and astrocyte‐neuron lactate shuttle (ANLS) proteins in wild‐type and hydroxycarboxylic acid receptor 1‐knockout (HCA1‐KO) mice with aging or long‐term L‐lactate infusion. In a mouse model of aging, neuronal FGF21 expression and ANLS rate were upregulated in hippocampal and cortical regions. Administration of exogenous FGF21 (1 mg/kg) to aging or lactate‐infused mice can significantly improve learning and memory performance and the lactate metabolic microenvironment in an MCT2‐dependent manner. Besides, HCA1‐KO can significantly abolish both the CREB and MAPK signaling activation in lactate‐infused mice, which differs from the scenario of aging mice. Furthermore, in vitro aging model further confirmed that p38‐mediated FGF21 production and PI3K‐mTOR‐dependent MCT2 protein translation process, respectively. The increase in levels of FGF21 protein as the brain ages might help neurons cope with age‐related neuroinflammation and lactate accumulation in mice. Our findings indicated that the shuttle rate of lactate and its microenvironment are related to neuronal function, which may be one therapeutic target for aging‐related cognitive dysfunction.

## Graphical abstract

The left panel shows cerebral lactate homeostasis in young mice. The middle panel displays accelerated ANLS rates due to FGF21 secretion in aging mice. The right panel demonstrates that lactate uptake and usage inhibition contribute to lactate extracellular accumulation, followed by HCA1‐mediated learning and memory decline in mice.

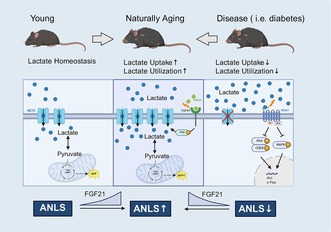

**Abbreviations**
AMPK: AMP‐activated protein kinase
ANLS: astrocyte‐neuron lactate shuttle
ERK: extracellular signal‐regulated kinase
FGF21: fibroblast growth factor 21
FGFR: fibroblast growth factor receptor
HCA1: hydroxycarboxylic acid receptor 1
MCT: monocarboxylic acid transporter
p38 MAPK: p38 mitogen‐activated protein kinase
PI3K: phosphatidylinositol 3‐kinase
ROS: reactive oxygen species
SA‐β‐gal: senescence associated‐beta‐galactosidase
SOD: superoxide dismutase
TNFα: tumor necrosis factor alpha

## 1 Introduction

Aging is an inevitable biological process characterized by a gradual decline in physiological cognitive functions, such as learning and memory (Yu et al. 2025; Lopez‐Otin et al. 2023; Ma et al. 2024). The intricate relationship between aging and cognitive deterioration has been a focal point of studies, with recent studies highlighting the role of metabolic regulation, i.e., lactate homeostasis (Magistretti and Allaman 2018; Frame et al. 2024). Lactate, once considered a mere waste product of glycolysis, is now recognized as a vital energy substrate and signaling molecule in the brain (Wang et al. 2024; Xue et al. 2022). The astrocyte‐neuron lactate shuttle (ANLS) hypothesis posits that astrocytes produce lactate, which is then transported to neurons to fuel oxidative metabolism and support synaptic activity (Pellerin and Magistretti 2012; Suzuki et al. 2011). Disruptions in this metabolic coupling have been linked to cognitive impairments associated with various neurodegenerative diseases (Yang et al. 2024), such as Alzheimer's disease (Newington et al. 2013) and Parkinson's disease (PD) (Lian et al. 2024).

In 2020, Sun et al. first reported that modulation of the ANLS system contributes to the ameliorated effects of fibroblast growth factor 21 (FGF21) on neurodegeneration of AD mice (Sun et al. 2020). FGF21, as a pro‐longevity hormone predominantly secreted by the liver and adipose tissues, has garnered attention for its pleiotropic effects on glucose and lipid metabolism, as well as the aging process (Szczepanska and Gietka‐Czernel 2022; Li et al. 2024; Sa‐Nguanmoo et al. 2016; Flippo and Potthoff 2021; Kang et al. 2021). Notably, FGF21 has been shown to enhance cognitive function in D‐galactose‐induced aging mice, possibly by improving energy metabolism and reducing oxidative stress (Yu et al. 2015). Besides, Zhang et al. reported that transgenic overexpression of FGF21 prolongs the lifespan of mice (Zhang et al. 2012). Exogenous FGF21 administration ameliorates aging‐related metabolic alterations such as insulin resistance and dyslipidemia in rodents (Taliyan et al. 2019; Morita et al. 2019). Also, it was reported that serum FGF21 levels were increased in healthy elderly individuals (Villarroya et al. 2018).

Our previous studies have found that FGF21 treatment can contribute to both learning and memory ability and lactate uptake in diabetic mice (Zhao et al. 2022). In the Parkinson's disease mouse model, we observed that FGF21 ameliorated motor and cognitive dysfunction via modulating lactate homeostasis (Yang et al. 2023, 2021). In high‐fat‐induced obese mice, the altered lactate homeostasis might lead to FGF21 resistance (Zhou et al. 2021). Furthermore, we confirmed that FGF21 enhanced learning and memory performance in mice by regulating lactate homeostasis (Xie et al. 2024; Zhang et al. 2024). Emerging studies suggest that FGF21 may influence lactate metabolism, following ameliorating cognitive function (Wang et al. 2025). Despite these advances, the causal relationship between endogenous FGF21 and lactate homeostasis in an aging environment remains poorly understood. Addressing this knowledge gap is crucial for developing therapeutic strategies to rescue age‐related cognitive decline.

In this study, we aim to elucidate the role of FGF21 in regulating lactate metabolism and its impact on cognitive function in naturally aged mice. By investigating the molecular pathways through which FGF21 influences lactate shuttling and the metabolic microenvironment, we aim to gain novel insights into the interplay between metabolic regulation and cognitive health during the aging process. Our findings may pave the way for new therapeutic interventions aimed at preserving cognitive function in the elderly.

## 2 Methods

### 2.1 Animals

Five sets of animal studies were performed: (1) measuring cerebral FGF21 and ANLS proteins in aging mice; (2) evaluating FGF21‐mediated protection from aging‐induced neuroinflammation and apoptosis; (3) assessing FGF21‐mediated cognitive protection from lactate homeostasis alteration induced by lactate infusion; (4) testing whether hydroxycarboxylic acid receptor 1‐knockout (HCA1‐KO) mice are susceptible to aging insult; and (5) testing whether HCA1‐KO mice are susceptible to lactate‐induced metabolic alteration. All animal experiments were performed in strict accordance with the National Institutes of Health Laboratory Animal Care and Use Guidelines and were approved by the Animal Health and Use Committee of Wenzhou Medical University (document number: wydw 2018–029).

Eight‐week‐old male C57BL/6 mice, procured from Shanghai Slack Laboratory Animal Co. Ltd., were housed in the SPF room of the Experimental Animal Center of Wenzhou Medical University. Subsequently, the mice were divided into three groups (*n* = 10 per group): young control group (2‐month‐old), aging group (20‐month‐old). Furthermore, the aging mice received either PBS or FGF21 (1 mg/kg, i.p.) daily for an additional 4 weeks (Figure 2A). The recombinant human FGF21 protein used in this study referred to our previous procedures (Zhou et al. 2024; Song et al. 2014). The animals were maintained under standard conditions of temperature and humidity, with a 12‐h light/dark cycle, and fed with a commercial normal chow diet and water. We took every precaution to minimize the number of mice used and the suffering of the animals in the study.

In another experiment, 1.5‐month‐old C57BL/6 mice were also randomly assigned into three groups: control, lactate, and lactate + FGF21 mice (*n* = 10 per group). For 12 consecutive weeks, each mouse was administered either PBS or lactate (1 g/kg, Sigma), following a previously described method (Kitaoka et al. 2016). Subsequently, the mice received either PBS or FGF21 (1 mg/kg, i.p.) daily for an additional 4 weeks (Figure 4A). The control group continued to receive PBS.

In the third set of experiments, C57BL/6 mice were randomly assigned into four groups: control, lactate, MCT2, lactate + MCT2 groups (*n* = 6 per group). Mice overexpressing monocarboxylate transporter‐2 (MCT2) were established using adeno‐associated virus‐PHP.eB (AAV‐PHP.eB) vectors, via tail vein injection. Mice in the negative control group received an equivalent volume of AAV‐PHP.eB‐NC virus. The virus was constructed and procured from Shanghai Heyuan Biology Co. Ltd.

HCA1‐KO mouse lines were obtained from Cyagen Biosciences (Taicang, China). The genotype of all animals used for the experiments was confirmed using polymerase chain reaction (PCR) analysis. In the study, 1.5‐month‐old WT and HCA1‐KO mice were randomized to control, 20‐month‐old aging, or lactate treatment for 12 weeks groups (*n* = 6 per group).

### 2.2 Adeno‐Virus Vector Stereotaxic Injection

2 and 20‐month‐old male mice were anesthetized with isoflurane and placed into a stereotaxic frame. Two small holes were bilaterally drilled in the skull (LH coordinates: AP = −0.46, ML = ±1.00 mm). 5 μL Neuro 7000 syringe (Hamilton) was filled with AAV (1.43 × 10^13^ virus genomes per μL) and the needle lowered into the holes to reach the LH (LH coordinates: DV = −2.40 mm). 2 μL of vector per hemisphere was injected at a speed of 0.4 μL/min using a Micro syringe Pump Controller Micro 4 (Harvard Apparatus). The mice received either pAAV‐U6‐NC‐CMV‐GFAP‐EGFP‐WPRE virus (NC Control or NC Aging mice) or pAAV‐U6‐shFGF21 (shMCT2)‐CMV‐GFAP‐EGFP‐WPRE virus (shFGF21 or shMCT2 Aging mice) to knockdown their expression in the mice.

### 2.3 Behavioral Tests

The Morris water maze (MWM) and Y‐maze tests were carried out as previously described (Zhao et al. 2011; Morris 1984). In brief, mice from each group were selected to perform learning tasks in these tests. Parameters such as swimming distance, the number of platform crossings, and time spent in the target object were recorded, respectively.

### 2.4 Histological Analysis

The tissues were isolated and fixed in 4% paraformaldehyde for at least 24 h. Thereafter, they were embedded in paraffin and sectioned using a slicing microtome (Leica, Germany). Nissl staining was conducted to detect brain morphology and neuron density using a Nissl Staining Kit (Beyotime, Shanghai, China) according to the manufacturer's protocol.

For the immunohistochemical staining experiments, paraffin sections were placed in an incubator at 60°C for 1 h. The dewaxed and dehydrated paraffin sections were then incubated in 3% H_2_O_2_ for 10 min and incubated in boiling citrate buffer for 5 min. Thereafter, the sections were blocked with 5% bovine serum albumin for 1 h. The paraffin sections were then incubated with the antibodies overnight at 4°C. After washing the sections in phosphate‐buffered saline (PBS), they were then incubated with the secondary antibodies at 37°C for 1 h. Images of the sections were captured using a Nikon ECLIPSE Ti microscope. For the immunofluorescence experiments, most protocols were similar to the immunohistochemical analysis. The only difference was that the sections were incubated with the fluorescent secondary antibody after washing in PBS.

### 2.5 Cell Culture and Treatment

Primary cortical neurons were cultured as previously described with some modifications (Kaech and Banker 2006). Briefly, cultures were prepared from Sprague–Dawley rat pups that were less than 1‐day‐old. Cerebral cortical tissues were dissected and the meninges were removed under a microscope. Cortical tissues were minced and digested using trypsin for 10 min at 37°C, and the trypsin was neutralized using DMEM medium containing 10% FBS. The cell suspension was passed through a 200‐μm mesh strainer to obtain single‐cell suspensions. Cells were plated on poly‐D‐lysine‐coated Falcon flasks at a density of 2 × 10^6^ cells/mL. Cells were cultured in a humidified incubator at 37°C for 12 h (Thermo Fisher Scientific, Waltham, USA). Cells were washed once with HBSS, and the medium was replaced with fresh Neurobasal serum‐free medium supplemented with B27, 2 mM glutamine, 100 U/mL penicillin, and 0.1 mg/mL streptomycin. 10 μM of Cytosine arabinoside was added and maintained for 12 h to prevent astrocyte proliferation. Primary neurons were cultured for 4 days, and the medium was changed twice weekly.

Primary astrocytes were cultured similarly to neurons' procedures with some modifications (McCarthy and de Vellis 1980). The cultures were also maintained in an incubator with 5% CO_2_ at 37°C. Then, the cell medium was changed after the first 3 days and twice a week thereafter. Upon reaching confluency, the microglia and oligodendrocytes were removed by an orbital shaker (220 rpm, for 18 h), then the purified astrocytes were passaged into new culture flasks.

The human undifferentiated neuroblastoma SH‐SY5Y cell line was purchased from ATCC and routinely maintained in DMEM media containing 10% FBS, 100 U/mL penicillin and streptomycin. The cell line was cultured in standard conditions (37°C, 5% CO_2_). To simulate in vitro cell aging, the cell line and primary neurons were treated with hydrogen peroxide (H_2_O_2_, Sigma–Aldrich) at 400 μM for 24 h. H_2_O_2_ was the inducer of cellular aging due to its widespread use and reliability in oxidative stress models.

The senescence‐associated‐beta‐galactosidase (SA‐β‐gal) staining solution was incubated for 48 h and observed with a light microscope for β‐galactosidase staining. Cells were incubated with 10 μM dihydroethidium (HDE, Beyotime Biotechnology, Shanghai, China) for 30 min in the dark. This incubation was done to analyze the levels of intracellular total reacting oxygen species (ROS), which were observed with a fluorescence microscope. Quantitative analysis was performed by using Image J software.

### 2.6 Enzyme Activity Assay

Lactate dehydrogenase (LDH) activity was measured using a protocol from our previous study (Zhao, Dong, Ren, et al. 2018). Briefly, we utilized an LDH assay kit from Jiancheng Bioengineering Institute (China), following the manufacturer's instructions.

### 2.7 Reverse Transcription‐Polymerase Chain Reaction (RT‐PCR) Analysis

Mouse cortical and hippocampal tissue samples were homogenized in TRIzol reagent (Invitrogen, Carlsbad, CA) to extract total RNA. The RT of RNA into cDNA was carried out using the PrimeScript Real‐Time Reagent Kit (Takara, RR037A). PCR analysis was conducted on a CFX96 Touch Real‐Time PCR Detection System (Bio‐Rad, Hercules, CA). The primer sequences are listed in Table S1 in the Supporting Information.

### 2.8 Western Blot Analysis

To extract total protein from tissues and cells, we used a buffer containing 1% protease and phosphatase inhibitors (Beyotime Biotechnology Institute). The protein samples were loaded onto an electrophoresis gel and then transferred to polyvinylidene fluoride membranes. After electrophoresis, the membranes were incubated in TBST for 2 h, then incubated overnight at 4°C with primary antibodies. Which includes: p‐JNK (1;1000; ET1609‐42, HUABIO), JNK (1:1000; ET1601‐28, HUABIO), p‐p38 (1:1000; 4511S, Cell Signaling Technology), p38 (1:1000; 8690S, Cell Signaling Technology), p‐Erk (1:1000; ET1610‐13, HUABIO), Erk (1:1000; ET1601‐29, HUABIO), SOD1 (1:1000; 10269‐1‐AP, Proteintech), SOD2 (1:1000; 24,127–1‐AP, Proteintech), NOX1 (1:1000; 17772‐1‐AP, Proteintech), NOX4 (1:1000; 14347‐1‐AP, Proteintech), β‐Actin (1:1000; 20536‐1‐AP, Proteintech), Bax (1:1000; 50599‐2‐Ig, Proteintech), Bcl‐2 (1:1000; ab59348, abcam), p‐mTOR (1:1000; 5536S, Cell Signaling Technology), mTOR (1:1000; 2983S, Cell Signaling Technology), p‐PI3K (1:1000; 17366S, Cell Signaling Technology), PI3K (1:1000; 4249S, Cell Signaling Technology), MCT2 (1:1000; 20355‐1‐AP, Proteintech), LDHA (1:1000; 21799‐1‐AP, Proteintech), LDHB (1:1000; 66425‐1‐Ig, Proteintech), FGF21 (1:1000; ET1704‐04, HUABIO), GAPDH (1:1000; 10494‐1‐AP, Proteintech), c‐Fos (1:1000; 2250S, Cell Signaling Technology), Arc (1:1000; 162901‐AP, Proteintech), p‐AMPK (1:1000; 2535S, Cell Signaling Technology), AMPK (1:1000; 5831S, Cell Signaling Technology), p‐Akt (1:1000; 4060S, Cell Signaling Technology), Akt (1:1000; 4691S, Cell Signaling Technology), MCT1 (1:1000; 20139‐1‐AP, Proteintech), MCT4 (1:1000; 22787‐1‐AP, Proteintech), p‐PKA (1:1000; 5661S, Cell Signaling Technology), PKA (1:1000; 5842S, Cell Signaling Technology), SAP97 (1:1000; HA721502, HUABIO), PSD95 (1:1000; ET1602‐20, HUABIO), FLAG (1:2000; 80010‐1‐RR, Proteintech), p53 (1:1000; 60283‐2‐lg, Proteintech), p21 (1:1000; HA722065, HUABIO), p16 (1:1000; HA601131, HUABIO), Caspase‐3 (1:1000; ab184787, Abcam), Cleaved Caspase‐3 (1:1000; ET1602‐47, HUABIO), p‐CREB (1:1000; ET7107‐93), CREB (1:1000; ET1601‐15, HUABIO), p‐Nrf2 (1:1000; DF7519, Affinity), Nrf2 (1:1000; AF0639, Affinity), SLC7A11 (1:1000; 26864‐1‐AP, Proteintech), GPX4 (1:1000; 67763‐1‐Ig, Proteintech). After washing the membranes three times with TBST, they were incubated with secondary antibodies (goat anti‐mouse IgG (H + L), 1:5000, SA00001‐1, Proteintech; goat anti‐rabbit IgG (H + L), 1:5000, SA00001‐2, Proteintech) at room temperature. The bands were detected using the ChemiDoc XRS^+^ Imaging System (Bio‐Rad), and the gray values of the bands were analyzed using Image J software.

### 2.9 Statistics

Data were presented as mean ± SEM. All data were analyzed using unpaired Student's *t*‐test and one‐way and two‐way analyses of variance (ANOVA) followed by post hoc test. Values of *p* < 0.05 were considered statistically significant. SPSS for Windows, Version 13.0 (SPSS Inc., Chicago, IL) was used for all statistical tests. The figures were generated using Prism 5.0 software (GraphPad Software Inc., San Diego, CA).

## 3 Results

### 3.1 Increased Astrocyte‐Neuron Lactate Shuttle (ANLS) and FGF21 Levels in Naturally Aging Brain

**FIGURE 1:**
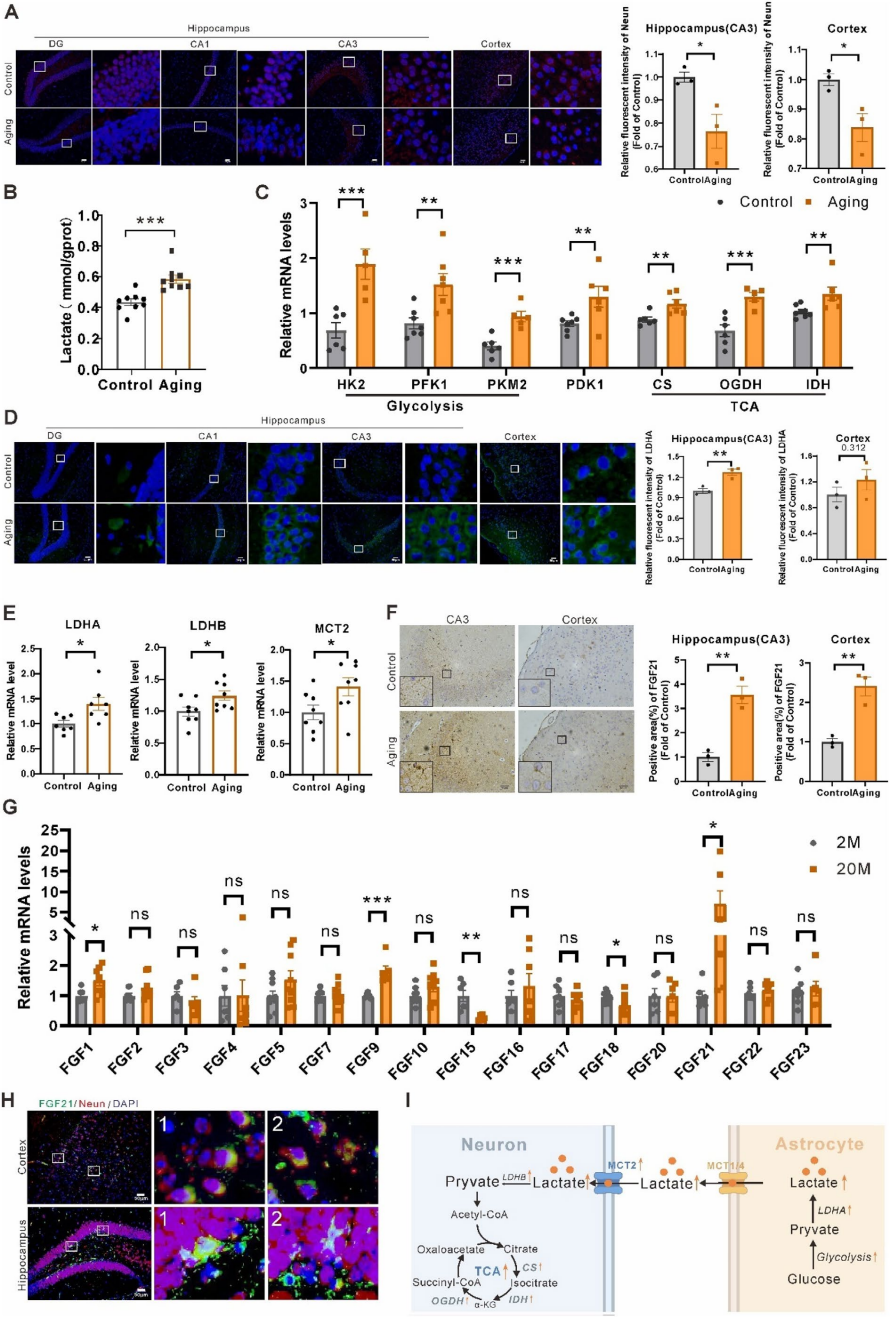
Increased cerebral FGF21 levels and astrocyte‐neuron lactate shuttling (ANLS) in naturally aging mice. (A) Immunofluorescence staining of NeuN and quantification in the hippocampus and cortex regions of aging (20‐month‐old) and young (2‐month‐old) mice (*n* = 3). (B) Lactate levels in cortical extracts of the mice (*n* = 9). (C) Graphical RT‐PCR quantification of enzyme levels related to glycolysis and TCA cycle in cortical extracts from the mice (*n* = 5–8). (D) Immunofluorescence staining of LDH‐A and quantification in the hippocampus and cortex regions of the mice (*n* = 3). (E) Graphical RT‐PCR quantification of enzyme levels related to lactate metabolism and shuttle in cortical extracts from the mice (*n* = 7–8). (F) Immunohistochemistry staining and quantification of FGF21 in the hippocampus and cortex regions of the mice (*n* = 3). (G) Graphical RT‐PCR quantification of FGF family members in cortical extracts of the mice (*n* = 6–10). (H) Colocalization of FGF21 (green), NeuN (red), and merge (yellow) in the cortex and hippocampus regions of the aging mice. (I) Schematic diagram of accelerated ANLS in the brains of aging mice (HK, hexokinase; PFK, phosphofructokinase; PK, pyruvate kinase; MCT, monocarboxylate transporter; LDH, lactate dehydrogenase; CS, citrate synthase; IDH, isocitrate dehydrogenase; OGDH, ketoglutarate dehydrogenase; NADH, nicotinamide adenine dinucleotide; PDK1, pyruvate dehydrogenase kinase 1). Data are presented as mean ± SEM. *p < 0.05, **p < 0.01, ***p < 0.001, determined by two‐tailed unpaired Student's *t* test. n.s., not significant. “*n*” represents the number of mouse samples in each group.

We evaluated the metabolic and inflammation status of hippocampal and cortical regions from young (2‐month‐old) and healthy aging (20‐month‐old) mice. Briefly, aged mice had significantly higher p53 levels than young mice (Figure S1A). Similarly, TUNEL staining revealed that aged mice exhibited more severe neuronal apoptosis than young mice (Figure S1B). By NeuN staining, the neuron number in the hippocampal and cortical regions of the aging mice was significantly decreased compared to controls (Figure 1A). Besides, in the aging mice, cortical AMPK and p38 MAPK phosphate levels, superoxide dismutase‐2 (SOD‐2), proto‐oncogene (c‐Fos), and activity‐regulated cytoskeletal‐associated protein (Arc) were all changed (Figure S1C). All the data indicate that there is manifest neuronal inflammation and dysfunction in the cortical and hippocampal regions, which may be potential risk factors for brain damage in aging conditions.

Interestingly, we found elevated lactate levels in hippocampal (Figure S1D) and cortical extracts (Figure 1B) from the aging mice, which were consistent with previous aging studies (Ross et al. 2010; Datta and Chakrabarti 2018). We interrogated whether lactate flux and usage differences also occur in the naturally aging brains. Both glycolytic enzymes (*hexokinase, HK; phosphofructokinase, PFK; pyruvate kinase, PK; pyruvate dehydrogenase kinase 1, PDK1*) and TCA cycle enzymes (*citrate synthase, CS; isocitrate dehydrogenase, IDH2; ketoglutarate dehydrogenase, OGDH*) mRNA levels were highly expressed in cortical (Figure 1C) and hippocampal regions (Figure S1E,F), suggesting a higher lactate production and metabolism phenotype in aging mice. The conversion of lactate to pyruvate and vice versa is catalyzed by the tetrameric complex of lactate dehydrogenases B and A (LDH‐B and A), respectively (Ross et al. 2010). We also observed LDH‐A is predominantly present in astrocytes whereas LDH‐B is predominant in neurons (Figure S1G,H). In aging brains, we found increased LDH‐A isoform expressions in the hippocampal and cortical regions by immunostainings, which reflected enhanced glial glycolytic activity (Figure 1D, Figure S1C). The higher mRNA levels of LDH‐B, LDH‐A, TCA cycle and MCT2 in hippocampal and cortical extracts further suggest elevated lactate metabolism and shuttle in aging brains (Figure 1E, Figure S1F).

Besides, we observed manifestly higher FGF21 protein levels in cortical and hippocampal regions, but not hepatic tissues (Figure 1F, Figure S2B). Interestingly, of all FGF members, FGF21 exhibited significant increases in cortical and hippocampal extracts (~5‐fold), as revealed by analyses with RT‐qPCR (Figure 1G, Figure S2A). Furthermore, colocalization of FGF21 and neural cells staining in cortical and hippocampal regions suggested that the increased FGF21 levels came mostly from neurons (marker: NeuN and DCX) rather than from astrocytes (marker: GFAP) and microglia (marker: IBA1) of the aging brains (Figure 1H, Figure S2C,D). Totally, the results indicated that elevated ANLS rates in aging mice acted in concert to regulate cerebral L‐lactate homeostasis (Figure 1I). These observations prompted us to investigate the causal relationship between FGF21 rise and lactate homeostasis in the aging mice's cerebral regions.

### 3.2 Exogenous FGF21 Administration Remits Learning and Memory Defects in Aging Mice

**FIGURE 2:**
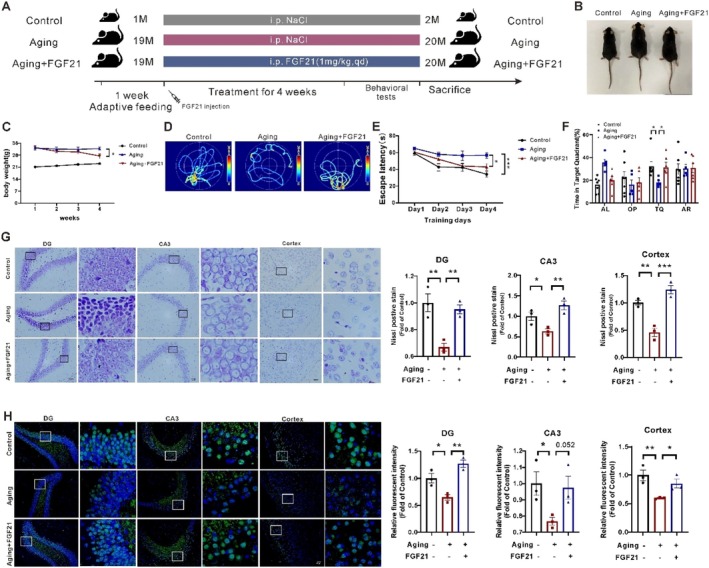
Exogenous FGF21 administration ameliorated learning and memory performance and neuronal loss in aging mice. (A) A schematic diagram illustrates the administration of FGF21 and PBS to the mice. (B) Macroscopic photos of the mice from the indicated group. (C) Body weights of the mice with different treatments (*n* = 5–7). (D–F) MWM test: Swimming trajectory, escape latency, percentage of time in the TQ of the mice (*n* = 5–7). (G) Representative images of Nissl body staining (blue spots, 200×) and quantification in hippocampal and cortical regions of mice (scale bar, 50 μm, *n* = 3). (H) Representative immunofluorescence images of NeuN staining (green dots, 200×) and quantification in the hippocampal and cortical regions of mice (scale bar, 50 μm, *n* = 3). Data are presented as mean ± SEM. *p < 0.05, **p < 0.01, ***p < 0.001, determined by one‐way ANOVA and followed by Turkey's multiple comparison test. n.s., not significant. “*n*” represents the number of mouse samples in each group. AL, Adjacent left. OP, opposite. TQ, target quadrant. AR, adjacent right.

To determine the therapeutic effect of FGF21 on the aging brain, we treated aging mice with 28 consecutive days of exogenous FGF21 (1 mg/kg) at 20‐month‐old (Figure 2A,B). Firstly, we compared the body weight and learning and memory performance of the aging and FGF21 mice, respectively. We found that FGF21 treatment for 4 weeks can significantly reduce the body weight of aging mice (Figure 2C). In the MWM test, compared to the control young mice, the aging mice showed a longer escape latency on the 4th day, as well as shorter platform crossing time and target quadrant time after removing the platform, suggesting impairments in their learning and spatial memory formation due to the aging insult. Nevertheless, the FGF21‐treated mice displayed distinct improvements in escape latency, platform crossing time, and target quadrant time compared to the untreated aging mice (Figure 2D–F). Overall, these results suggest that FGF21 administration can alleviate learning and memory defects in aging mice.

Besides, both Nissl and NeuN staining results revealed fewer Nissl bodies and neuronal numbers, respectively, in the hippocampus (DG and CA3 regions) and cortex of the aging mice. Conversely, the neuronal loss condition was not observed in FGF21‐treated aging mice (Figure 2G,H). These results demonstrated that FGF21 can also alleviate neuronal apoptosis or death in the brains of the aging mice, a finding that supports the restored behavioral performance.

### 3.3 FGF21 Treatment Is Associated With Reduced MAPK Signaling and Upregulation of ANLS‐Related Proteins in the Aging Brain

**FIGURE 3:**
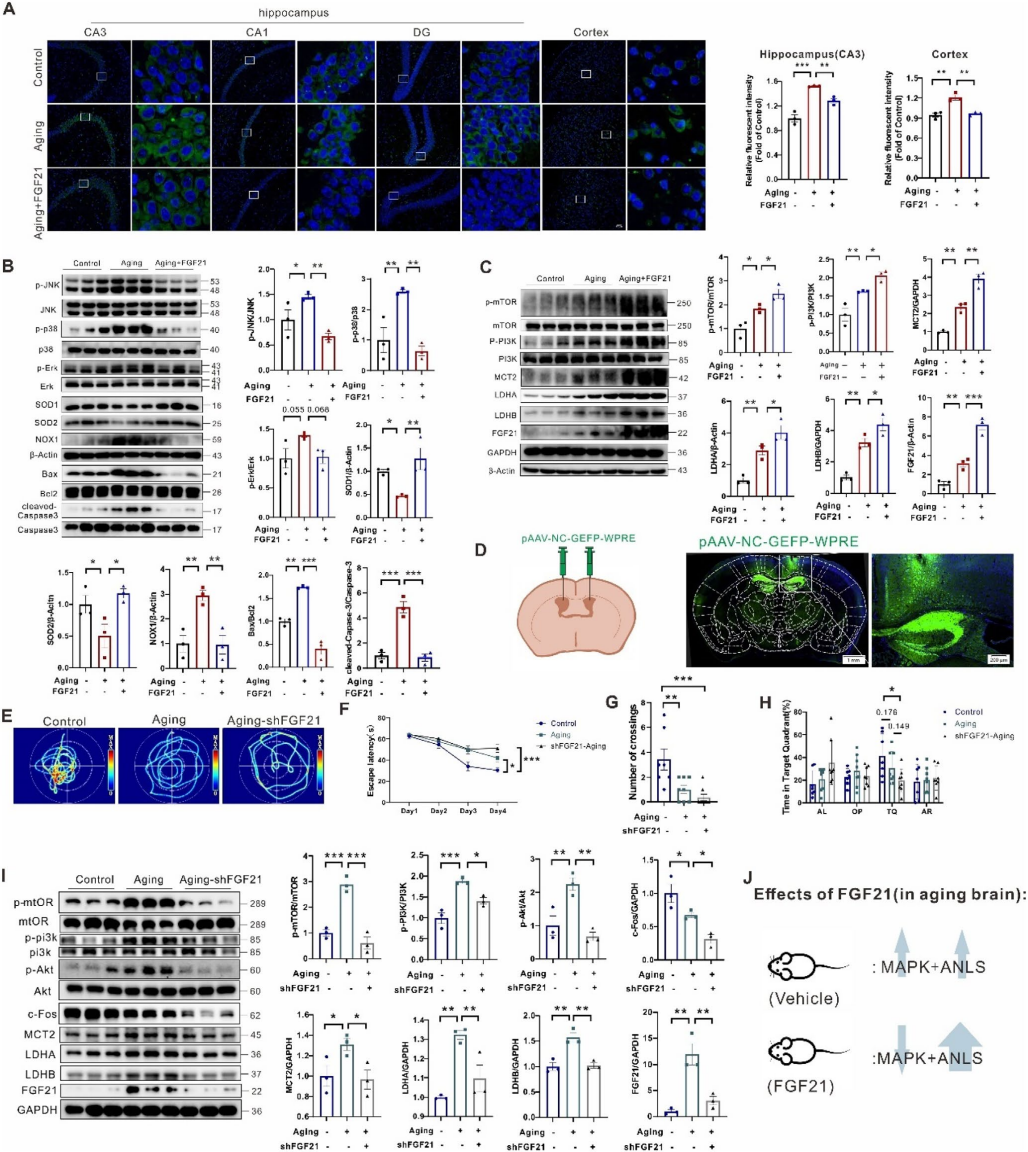
FGF21 attenuated MAPK‐apoptotic signals but accelerated PI3K‐mTOR and ANLS pathways in aging brain. (A) Immunofluorescence staining of TNF‐α in the hippocampus and cortex regions of the mice (*n* = 3). (B) Representative western blots and densitometric quantifications of MAPK, oxidative stress, and apoptosis signaling in the cortical extracts from the mice (*n* = 3). (C) Representative western blots and densitometric quantifications of mTOR‐PI3K and lactate metabolism pathways in the cortical extracts from the mice (*n* = 3). (D) A schematic diagram illustrates the strategy for stereotaxical adenovirus injections at the indicated locations in the aging mice. (E–H) MWM test: Escape latency, path length, platform crossing, percentage of time spent in the TQ of mice with different treatments (*n* = 7–8). (I) Representative western blots and densitometric quantifications of mTOR‐PI3K and lactate metabolism pathways in the cortical extracts from the mice (*n* = 3). (J) Schematic diagram of targets of FGF21 in the aging brains. Data are presented as mean ± SEM. *p < 0.05, **p < 0.01, ***p < 0.001, determined by one‐way ANOVA followed by Turkey's multiple comparison test.

To explore the mechanisms of FGF21's effect on the aging brain, we examined the neuroinflammation and apoptotic markers in the brains of the mice. Firstly, microscopy showed that exogenous FGF21 administration could manifestly decrease TNF‐α‐positive cells in hippocampal and cortical regions of the aging mice, suggesting the agent could restore the neuroinflammation in aging conditions (Figure 3A). Neurons possess a variety of antioxidant enzymes and non‐enzymatic antioxidant molecules that help combat oxidative stress and neuroinflammation. As aging progresses, these antioxidant defense mechanisms may become weakened, leading to increased oxidative stress. Then, aging also significantly increased cortical MAPK signaling (i.e., MAP kinases JNK and p38 phosphorylation) and prooxidant NADPH oxidase‐1 (NOX1), Bax expressions, cleaved‐caspase‐3, and decreased antioxidant superoxide dismutase‐1, 2 (SOD1, 2) and Bcl‐2 expressions (Figure 3B, Figure S3), all of which were completely prevented by FGF21. These results suggest that FGF21 prevents aging‐induced neuronal apoptosis in cortical regions via decreasing JNK and p38 MAPK phosphorylation and oxidative stress pathways.

We further compared the causal relationship between FGF21 and ANLS‐related protein expressions. We first confirmed the enhancement of neuronal lactate uptake and usage protein expressions in aging brains (Figure 3C). Interestingly, FGF21 administration did not restore but further increase the PI3K and mTOR phosphorylation, as well as MCT2, LDHA, and LDHB protein expressions in cortical extracts compared to aging mice, suggesting higher ANLS rate was driven by FGF21 in a PI3K‐mTOR dependent manner, which was also confirmed by the gradient increase of FGF21 level itself (Figure 3C). Secondly, since FGF21 is the upstream factor for ANLS rate, FGF21 deficiency could logically lead to decreased ANLS‐related protein levels and learning and memory impairment in aging mice. Therefore, stereotaxic adenovirus injections were performed at the indicated locations to quantify learning and memory performance and lactate metabolic protein levels in aging mice with reduced FGF21 expression. Green fluorescence in the hippocampal and cortical area 2 weeks post‐injection confirmed successful adenovirus transduction (Figure 3D). In the MWM task, the aging mice without FGF21 expression exhibited longer escape latencies and fewer platform crossings, as well as less time spent in the platform quadrant compared to normal aging mice (Figure 3E–H). Western blot analysis confirmed significant reductions in c‐Fos expression in cortical extracts, indicating a trend toward learning and memory inhibition. Besides, FGF21 reduction partially alleviated mTOR‐PI3K signal phosphorylation and lactate metabolism and transport protein levels, suggesting FGF21 as an upstream factor of lactate metabolism (Figure 3I). Collectively, these results demonstrate that FGF21 ablation significantly inhibits MCT2 expression, thereby contributing to decreased learning and memory performance in aging mice.

Furthermore, stereotaxic adenovirus injections of sh‐MCT2 were also applied at the indicated locations to quantify learning and memory performance and lactate metabolic protein levels in aging mice (Figure S4A). In the MWM task, the aging mice with reduced MCT2 expression also exhibited longer escape latencies and fewer platform crossings, as well as less time spent in the platform quadrant, compared to normal aging mice (Figure S4B–E). Western blot analysis displayed significant reductions in c‐Fos expression in cortical extracts, indicating a trend toward learning and memory inhibition. Interestingly, MCT2 reduction reversed ANLS‐related protein levels, but not mTOR‐PI3K activity, suggesting MCT2 as an upstream factor of ANLS, but not the mTOR signal (Figure S4F). In summary, these findings indicate that upregulation of ANLS‐related proteins may be involved in therapeutic mechanisms of FGF21 in aging brains (Figure 3J).

### 3.4 FGF21 Administration Rescued MAPK Signaling and ANLS Pathway in the Mice With Long‐Term Lactate Exposure

**FIGURE 4:**
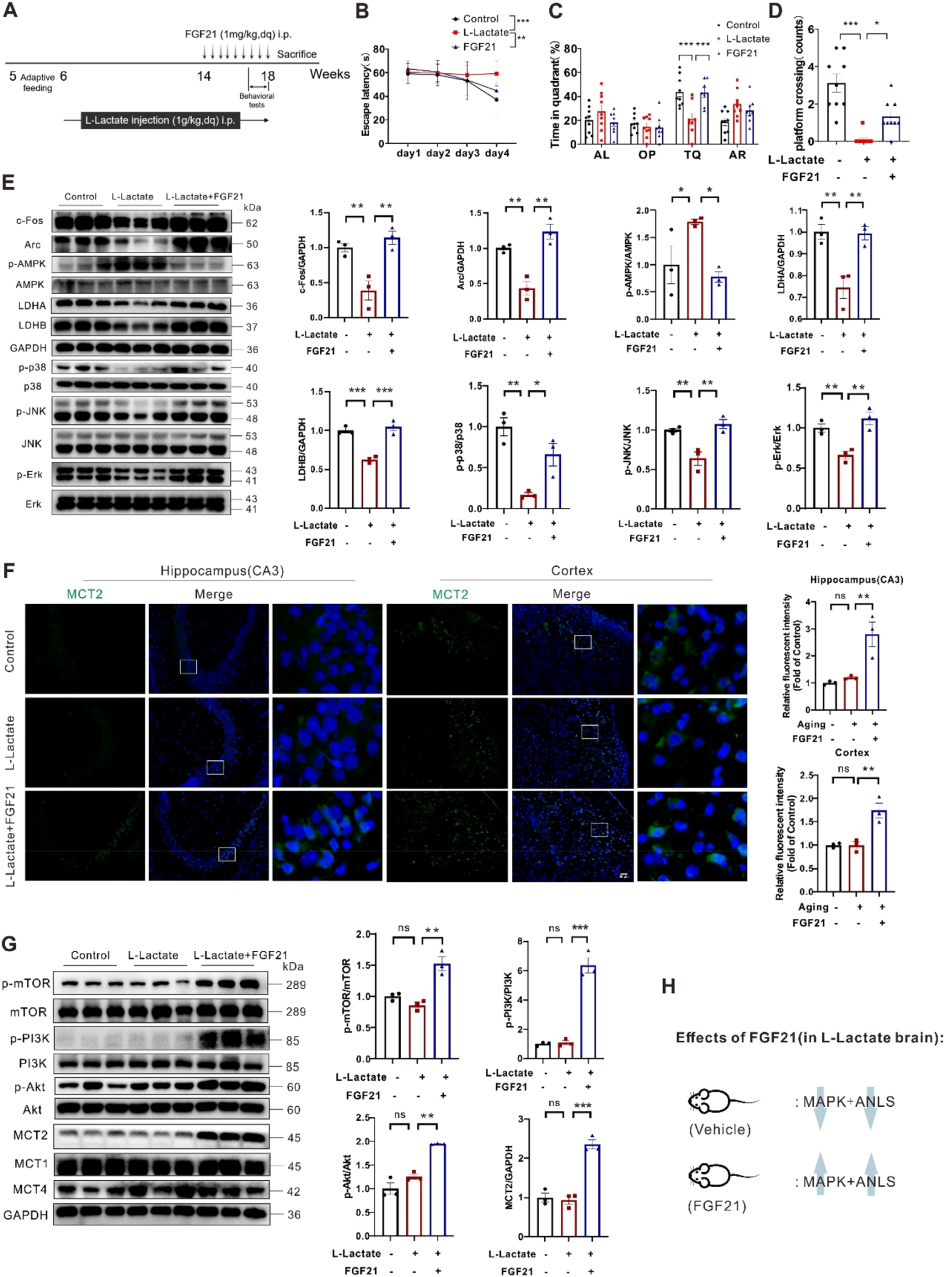
FGF21 administration rescued MAPK and ANLS pathways but enhanced PI3K signaling in the mice with long‐term lactate exposure. (A) A schematic diagram of lactate and FGF21 administration for mice. (B–D) MWM test: Escape latency, platform crossing, percentage of time in the TQ of the mice (*n* = 7–9). (E) Representative western blots and densitometric quantifications of mTOR–Akt signaling and lactate transport and metabolism proteins in the cortical extracts from the lactate and FGF21 mice (*n* = 3). (F) Immunofluorescence staining and quantification of MCT2 expression in the hippocampus and cortex regions of the mice (*n* = 3). (G) Representative western blots and densitometric quantifications of AMPK and MAPK (JNK, ERK, and P38) activity in the cortical extracts from the mice (*n* = 3). (H) Schematic diagram of the target of FGF21 in the brains of mice with long‐term lactate exposure. Data are presented as mean ± SEM. *p < 0.05, **p < 0.01, ***p < 0.001, determined by one‐way ANOVA followed by Turkey's multiple comparison test.

To further determine the causal relationship between ANLS and FGF21, and explore if the PI3K/mTOR/MCT2 axis is involved in the effects of FGF21 on learning and memory ability, we studied the actions of the agent under the circumstance of attenuated ANLS. According to our previous studies, through perturbative sodium lactate infusion for 90 consecutive days, we established a lactate‐accumulated mouse model with attenuated ANLS rate (Zhao et al. 2022; Haowei Jiang et al. 2021). To evaluate the therapeutic effects of FGF21 in this condition, we also treated the mice with FGF21 for 28 days and monitored the behavioral and related molecular changes (Figure 4A). In the Y‐maze test, we found that FGF21 treatment manifestly ameliorated the rate of spontaneous alternation (Figure S5A,B). In the MWM test, the escape latency of FGF21‐treated mice was significantly decreased compared to lactate mice. During the probe trial period on the 5th day, FGF21 mice also showed an increased platform crossing in the target quadrant (Figure 4B–D). Besides, we examined the mRNA levels underlying the process of learning and memory, such as *c‐Fos*, *Arc*, *early growth response 1* (*Egr‐1*), and *postsynaptic protein‐93* (*PSD93*) in cortical extracts from the mice. The results also showed that FGF21 administration could restore their levels (Figure S5C–G). Similar to aging conditions, the data indicated that FGF21 administration can also ameliorate learning and memory performance in mice with long‐term lactate exposure. Then, we further determined the dose‐effect and time‐effect of FGF21 on lactate metabolic protein and bioenergetic levels of SH‐SY5Y cells. Interestingly, we observed that 100 ng/mL of FGF21 can time‐dependently elevate MCT2 (but not MCT1 or 4) and LDH protein levels, as well as p‐AMPK levels, up to 24 h after treatment (Figure S5H). Similarly, FGF21 can also dose‐dependently increase these protein levels, up to 150 ng/mL (Figure S5I). The data suggested that the effects of FGF21 on learning and memory may be related to upregulation of ANLS‐related proteins.

Analogously, we further observed that FGF21 can also ameliorate reduced cortical IEG (Arc and c‐Fos) protein levels due to long‐term lactate exposure, which is consistent with the results of behavior tests (Figure 4E). Besides, the MAPK signaling, including ERK, p38 MAPK, and JNK phosphorylation, was all inhibited in lactate accumulation conditions (Figure 4E), which is opposite to the aging brains, indicating the MAPK signaling activation is the result of aging, but not lactate accumulation insult. Nevertheless, we also found that FGF21 treatment can restore the MAPK signaling activity in cortical extracts (Figure 4E). Inconsistent with aging brains, we observed a greater increase in p‐AMPK (bioenergetic sensor (Jiang et al. 2018)) and decreases in LDH‐A and LDH‐B levels in the lactate mice, which reflect decreased bioenergetic levels and ANLS rate. These attenuations can also be reversed by FGF21 treatment (Figure 4E). Interestingly, we observed that increased PI3K, mTOR, and Akt phosphorylation levels and MCT2 protein expressions only in FGF21 mice, but not lactate and control mice (Figure 4F,G), further confirmed PI3K‐Akt–mTOR pathway was involved in the mechanism of FGF21 on ANLS rate and MCT2 expressions, which are consistent with the results of aging mice. Overall, these results suggest that FGF21 may prevent learning and memory impairment caused by long‐term lactate exposure by activating MAPK signaling and the MCT2‐dependent ANLS rate (Figure 4H).

### 3.5 Extracellular Lactate Accumulation Attenuated Both Learning and Memory Performance and MAPK Signaling Activity in a HCA1‐Dependent Manner

To study the lactate flux characteristics of neurons and astrocytes, we established an in vitro model to determine lactate uptake and release (Figure S6A). Unexpectedly, we found inhibited uptake of lactate in primary neurons after exogenous lactate exposure in a dose‐dependent manner (Figure S6B). Then, MCT2 silencing by shRNA or pharmacological inhibitor (AR‐C155858, 2 μM) can also inhibit lactate uptake in neurons (Figure S6C,D). Besides, we observed no decline in lactate uptake in neurons after FGF21 treatment. Next, we found manifestly increased lactate release rate from primary astrocytes, but not from neurons, in a high glucose environment (Figure S6E,F). This suggests that lactate exposure can significantly inhibit the neuronal uptake rate of lactate in an MCT2‐dependent manner. As a result, chronic lactate exposure may lead to extracellular lactate buildup in the metabolic microenvironment.

We next examined whether HCA1 (hydroxycarboxylic acid receptor 1, also known as GPR81) activation was involved in the different lactate metabolic microenvironment in aging and lactate mice, respectively. HCA1 is a lactate‐activated G‐protein‐coupled receptor predominantly expressed on neuronal plasma membranes (de Castro Abrantes et al. 2019). Upon binding the extracellular lactate ligand, HCA1 signals via inhibitory Gi protein, suppressing cAMP‐CREB activity in fat cells (Liu et al. 2009). Thus, we investigated whether HCA1 receptor activation could modulate properties of lactate involved in the PKA‐CREB pathway. Our data showed that application of both lactate (10 mM) and an agonist of HCA1 receptor, namely 3,5‐DHBA (300 μM), caused decreases in pCREB levels on primary neurons, respectively (Figure S7A). Then, the inhibitory effect of lactate was reversed by forskolin (20 μM, activator of adenylate cyclase) on primary neurons (Figure S7B), suggesting the inhibitory effect operated by lactate was dependent on cAMP‐CREB activity. Besides, the declined phosphorylation levels of PKA and CREB by lactate treatment were also found in HEK293 cells with HCA1 overexpression, but not in the negative cells (Figure S7C). Interestingly, we found significantly inhibited PKA‐CREB activity in lactate‐infused mice, but not in aging mice (Figure S7D).

**FIGURE 5:**
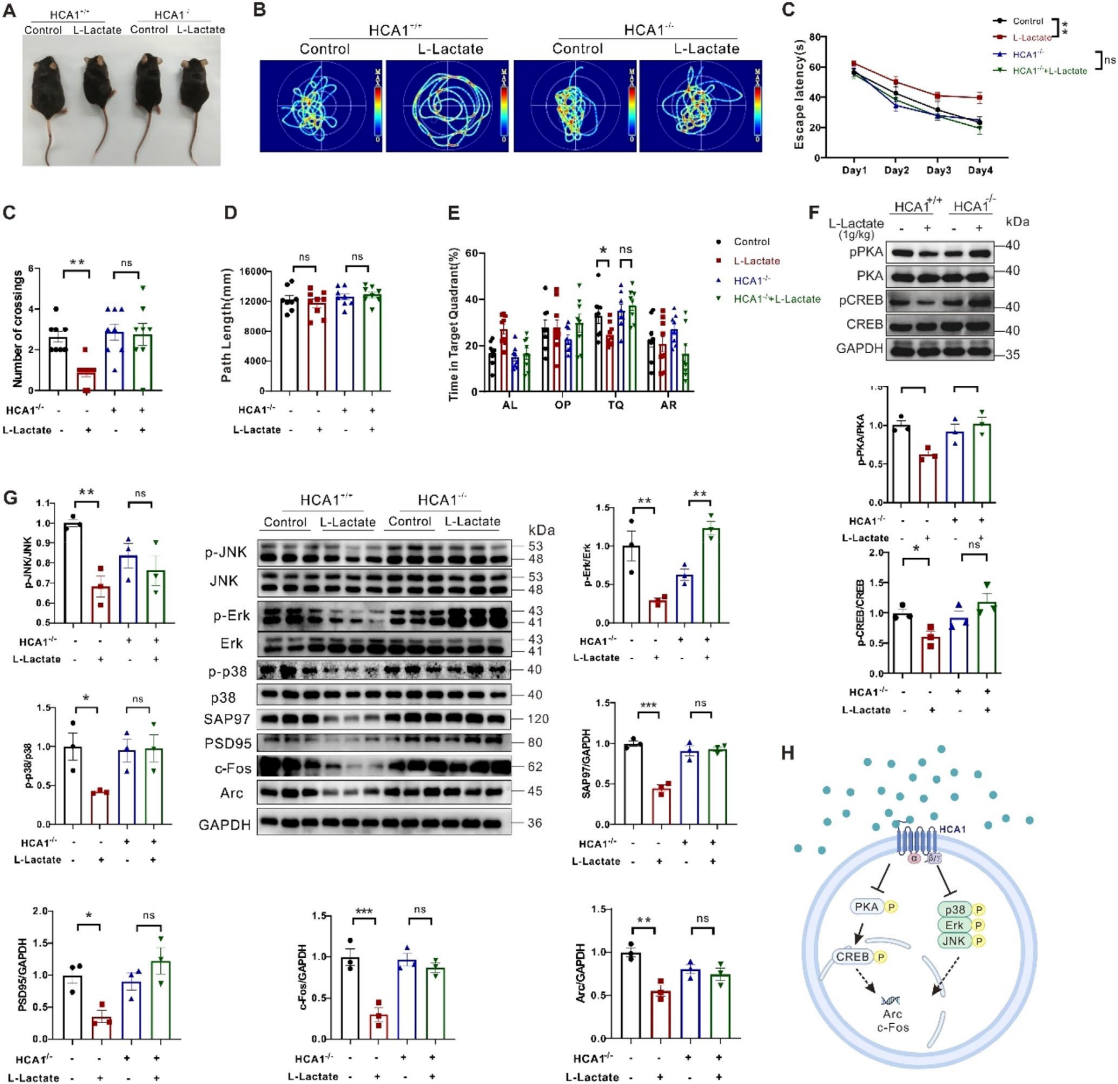
Long‐term lactate administration attenuated learning and memory performance, CREB and MAPK signaling pathways in an HCA1‐dependent manner. (A) Macroscopic photos of the mice after a 90‐day infusion of perturbative quantities of sodium‐lactate to HCA1 wild‐type (WT) or knockout (KO) mice. (B‐E) MWM test: Escape latency, path length, platform crossing, percentage of time in the TQ of the mice (*n* = 8–9). (F) Representative western blots and densitometric quantifications of PKA and CREB phosphorylation in the cortical extracts from the mice (*n* = 3). (G) Representative western blots and densitometric quantifications of JNK, ERK, and p38 MAPK phosphorylation, synaptic proteins, and IEG levels in the cortical extracts from the mice (*n* = 3). (H) Schematic diagram of HCA1‐mediated MAPK and CREB signaling pathways in the brains of mice with long‐term lactate exposure. ns, not significant. Data are presented as mean ± SEM. *p < 0.05, **p < 0.01, and ***p < 0.001 determined by two‐way ANOVA and followed by Dunnett multiple comparison test.

To ascertain whether the effect of lactate on learning and memory ability was via the HCA1‐dependent CREB pathway, we first performed perturbative lactate infusion in HCA1 knockout (KO) mice and compared it with that from the wild‐type (WT) mice (Figure 5A, Figure S8A–C). After an 84‐day lactate infusion, in the Y‐maze test, the rate of spontaneous alternation was significantly reduced in WT mice compared with controls, whereas HCA1‐KO mice remained unchanged (Figure S8D,E). In the MWM test, the escape latency, number of crossings, and time in the target quadrant indicators were also reduced in WT mice, but not KO mice (Figure 5B–E). And the PKA‐CREB signaling alteration is similar to the behavior performance (Figure 5F), which indicates that lactate suppressed learning and memory performance in the mice was dependent on HCA1‐PKA‐CREB activity, which is consistent with our previous diabetic studies (Zhao, Dong, Ren, et al. 2018). Similarly, we also found that the protein levels of Arc, c‐Fos, PSD95, SAP97, as well as MAPK signaling, were all reduced in lactate‐treated WT mice, but not HCA1 absent mice (Figure 5G). These findings suggest that HCA1‐KO can significantly abolish both the CREB and MAPK signaling activation in lactate‐accumulated mice, which may participate in the mechanism of effects of lactate exposure on learning and memory (Figure 5H).

We next examined whether HCA1 activation is also involved in aging brains. The HCA1‐KO mice were aged to 20 and 2 months, respectively, and cortical FGF21 and ANLS‐related protein expressions were examined by WB (Figure S8F). In contrast to lactate brains, aging induced increases in FGF21, p‐AMPK, MCT2, and LDHB levels in both wild‐type and HCA1 KO mice, suggesting that the effects of lactate in aging brains are independent of HCA1 activation. Therefore, HCA1 activation is involved in lactate‐exposed brains, which differs from the scenario of aging‐related ANLS rate acceleration and increased FGF21 levels. Besides, the MAPK signaling was activated in aging brains but inhibited in the lactate mice. All the data suggested that the lactate metabolic microenvironment of aging brains has the feature of accelerated ANLS rate, which is different from that of lactate mice in which HCA1 is activated.

### 3.6 FGF21 Modulates Neuronal Lactate Uptake to Counter Cellular Senescence via an MCT2‐Dependent Manner

**FIGURE 6:**
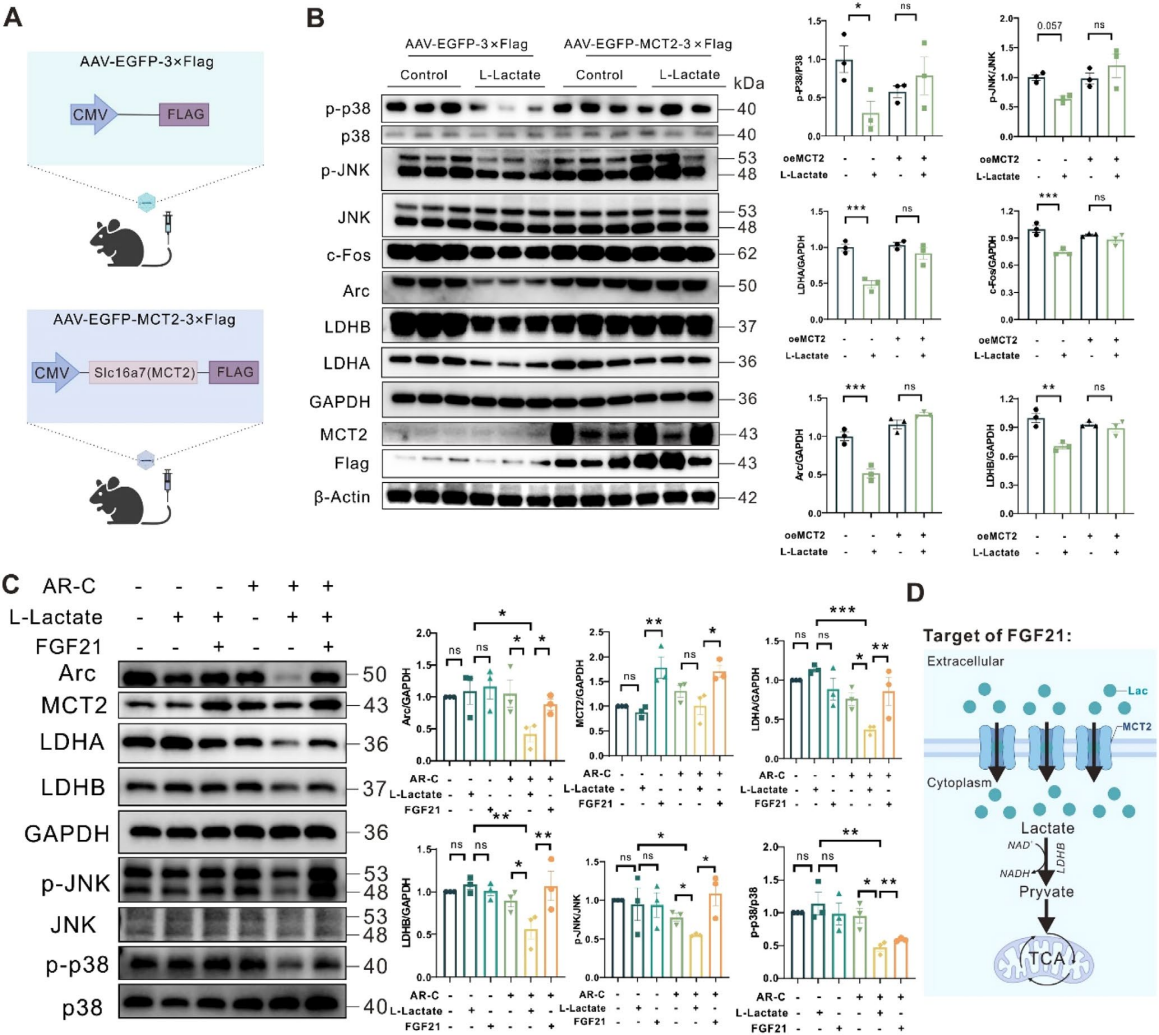
FGF21 Contributes to neuronal lactate uptake in an MCT2 overexpression manner. (A) Schematic diagram of *slc16a7* (MCT2)‐FLAG plasmid of AAV‐MCT2 or AAV‐NC. (B) Representative western blots and densitometric quantifications of Arc, c‐Fos, LDH‐A/B, and MAPK pathways phosphorylation levels in the cortex of AAV‐MCT2 or AAV‐NC mice with or without lactate treatment. β‐Actin was used as a loading control (*n* = 3). (C) SH‐SY5Y cells were treated with or without an MCT2 inhibitor (AR‐C155858, 1 μM) for 1 h before lactate stimulation for 24 h (*n* = 3). Representative western blots and densitometric quantifications of Arc, LDH‐A/B, and MAPK activity phosphorylation levels in cells. (D) Schematic diagram of the target of FGF21 in the brains of mice with lactate exposure. Data are presented as mean ± SEM. *p < 0.05, **p < 0.01, ***p < 0.001, determined by two‐way ANOVA and followed by Dunnett multiple comparison test.

Because MCT2 is a rate‐limiting barrier for cellular metabolism and function (Felmlee et al. 2020; Perez‐Escuredo et al. 2016; Song et al. 2020). To identify whether MCT2 is involved in a causal relationship of lactate uptake and FGF21, mice were infected by adeno‐associated virus type PHP.eB (AAV‐PHP.eB) containing mouse *SLC16A7* cDNA (MCT2) with and without lactate for 84 days (Figure 6A). Overexpression of cortical MCT2 completely abolished the decreased ERK, JNK, and p38 MAPK phosphorylation as well as LDH‐A and LDH‐B levels in lactate‐infusion mice (Figure 6B), which mimicked the effects of FGF21 in the brains. Additionally, in SH‐SY5Y cells, by adding exogenous lactate and an MCT2 blocker (AR‐C155858, 1 μM) to the media, we created an extracellular lactate environment (Erlichman et al. 2008). Firstly, decreases of JNK and P38 MAPK phosphorylation, as well as LDH‐A and LDH‐B levels in the cells were observed after lactate and AR‐C155858 treatment (Figure 6C), which mimicked the effects of lactate exposure in the brain. Then, the attenuated effects were significantly abolished by FGF21 treatment, which could also increase the MCT2 levels (Figure 6C). Manifestly, FGF21 incubation blunted the inhibitory effect of lactate on the MAPK activity and ANLS rate, suggesting the therapeutic effects of lactate on neurons were related to neuronal lactate uptake and MCT2 overexpression (Figure 6D).

To define the remarkable induction of neuronal FGF21 expression and protective effects at the stage of aging as a protective response to oxidative stress, SH‐SY5Y cells and primary neurons were treated with H_2_O_2_ to mimic cellular senescence, respectively. Exposure of the cells to H_2_O_2_ at 400 μM for 24 h, followed by SA‐β‐gal assays, immunofluorescence, and western blot analysis (Figure S9A). Firstly, the data showed that the SA‐β‐gal‐positive cells were significantly increased after H_2_O_2_ treatment, indicating that H_2_O_2_ treatment induced cell senescence (Figure S9B). Western blot results demonstrated a significant increase in the levels of p53, p21, and p16 protein in groups treated with H_2_O_2_, resulting in a more pronounced DNA damage response in SH‐SY5Y (Figure S9C). Besides, compared to the control group, H_2_O_2_ treatment significantly decreased antioxidant enzyme activities (SOD1, SOD2) while increasing NOX4 levels, a marker of prooxidant protein. Consistently, the Bax/Bcl‐2 ratio was also more evident in the cell senescence (Figure S9C). These findings indicate that the activation of oxidative stress and apoptosis occurs in the in vitro model of the aging model of neurons. Besides, representative dihydroethidium (DHE) fluorescence images and semi‐quantitative statistical results indicated elevated levels of ROS in H_2_O_2_‐treated SH‐SY5Y cells (Figure S9D).

We further investigated whether the expression of FGF21 increased in H_2_O_2_‐treated primary neurons. At 24 h posttreatment, immunofluorescence and western blot data (Figure S10A,B) demonstrated that compared to the control group, there was an elevation in FGF21 protein expression in primary neurons treated with H_2_O_2_. The mechanistic study revealed that H_2_O_2_ also stimulated the phosphorylation of ERK1/2, p38 MAPK, and JNK (Figure S10C). To establish whether the upregulation of these kinases is required for H_2_O_2_‐mediated FGF21 production, the primary neurons were treated with both H_2_O_2_ (400 μM) and an inhibitor of p38 MAPK (SB1290, 10 μM) and JNK (SP600125, 20 μM) for 2 h or ERK1/2 (PD98059, 20 μM) for 24 h before and during H_2_O_2_ treatment for 24 h (Figure S10C). The H_2_O_2_‐mediated FGF21 production was only attenuated by the p38 inhibitor, suggesting p38‐mediated FGF21 production in the aging model of neurons; it also supported the fact of involvement of oxidative stress mechanisms, like p38 elevation as the cause of FGF21 rise in two brain regions (cerebral cortex and hippocampus) of aging mice.

Our present and previous studies found that the therapeutic target of FGF21 was MCT2 expression via PI3K/AKT/mTOR signal activity (Zhao et al. 2022). This is consistent with other factors, such as brain‐derived neurotrophic factor (BDNF) (Robinet and Pellerin 2010), and insulin growth factor‐1 (IGF‐1) (Chenal et al. 2008). Additionally, it has been reported that in skeletal muscle, FGF21 can increase MCT2 expression (Yano et al. 2022). To further validate the role of the PI3K pathway on FGF21‐mediated MCT2 expression, we used a PI3K inhibitor (LY294002, 20 μM) and mTOR inhibitor (Rapamycin, 20 nM) to treat primary neurons. By immunofluorescence staining, we found that activation of MCT2 expression by FGF21 in neurons was markedly compromised by the inhibitors (Figure S10D). This in vitro model further strengthened the notion that the PI3K‐mTOR pathway was involved in the effects of FGF21 on MCT2 expression under aging conditions (Figure S10E).

## 4 Discussion

Aging is a significant risk factor for AD, in which metabolic dysfunction is recognized as a feature (Drulis‐Fajdasz et al. 2018). In fact, AD is also considered type 3 diabetes, a viewpoint that emphasizes the critical role of altered brain metabolism in AD (Sun et al. 2020; Leszek et al. 2017). Besides, aging is also characterized by chronic inflammation, commonly called inflammaging, which manifests as a low‐grade, persistent proinflammatory state (Xie et al. 2025). Our findings provide the first evidence linking FGF21 to lactate homeostasis in the aging brain. We identified multiple antiaging effects of FGF21, including the reduction of neuronal apoptosis, protection against oxidative stress, and mitigating inflammaging. Furthermore, our data showed that lactate extracellular accumulation is detrimental to neuronal metabolism and function. As a result, the anti‐AD‐like effects of FGF21 rely more on accelerating lactate uptake and utilization, and ameliorating the lactate metabolic microenvironment.

Our observations displayed a causal relationship between lactate microenvironment and neuronal activity in aging brains. The elevated lactate production by LDH‐A in astrocytes and the higher lactate uptake capacity in neurons suggest an enhanced metabolic interaction between astrocytes and neurons in the hippocampal and cortical regions. This also prompted us to investigate the attenuated ANLS conditions with long‐term lactate administration. Our data revealed an important role for elevated lactate levels, characteristic of the Warburg effect, in regulating neuronal metabolism and function. Interestingly, multiple previous studies have shown that exogenous lactate is beneficial for learning and memory (El Hayek et al. 2019; Scavuzzo et al. 2020; Fernandez‐Moncada et al. 2024; Wu et al. 2024), in contrast to our data showing that chronic lactate injections interfere with learning and memory. Bozzo et al. first reported that lactate diminishes neuronal activity (Bozzo et al. 2013). Subsequently, they found that a reduction in HCA1‐dependent spiking activity correlates with the effects of lactate (Wang et al. 2019). We recognized that the duration of lactate exposure and the state of lactate accumulation might be involved in the dual effects of lactate on learning and memory. By increasing lactate uptake and utilization, FGF21 might contribute to the neuronal bioenergetic metabolic homeostasis and cognitive amelioration in mice, particularly with aging. The directionality of lactate transport is primarily defined by the lactate concentration gradient across the cell membrane, in which MCT2 maintains extracellular basal lactate homeostasis (Xie et al. 2024). After uptake, lower lactate levels in neurons are also maintained by serving as a substrate for mitochondrial OXPHOS. Extracellular lactate accumulation can also bind the HCA1 to trigger a myriad of responses in cells (de Castro Abrantes et al. 2019). Furthermore, lactate may induce lactylation of histones and non‐histone proteins, an aspect that warrants our further investigation in the future (Nguyen et al. 2025). Manifestly, lactate metabolic microenvironment may become a potential target for the treatment of neurodegenerative diseases.

Neurons express HCA1, but their role in neuronal function, especially in aging circumstances, is almost unexplored (de Castro Abrantes et al. 2019; Lauritzen et al. 2014). In this study, the present data demonstrated that deletion of HCA1 receptor does not alter basal lactate metabolism induced by aging insult, suggesting HCA1 is not an upstream factor of lactate homeostasis in aging brains. However, our present and previous data altogether displayed that activation of HCA1 receptor by direct lactate treatment results in a time‐dependent inhibition of learning and memory performance in mice (Zhao, Dong, Ren, et al. 2018; Zhao, Dong, Wang, et al. 2018). We and others showed that these stimuli can trigger a plethora of intracellular signals, including attenuated CREB phosphorylation and MAPK signal activity in mice, which could interact with HCA1 receptor on the cellular membrane (Zhao et al. 2022; Wang et al. 2019; Lee et al. 2025; Briquet et al. 2022; Li et al. 2014; Yan et al. 2023). Conversely, in the aging mice, we found that increasing JNK and p38 MAPK phosphorylation and PI3K‐mTOR mediated lactate enzyme and transporter elevations, but HCA1 was not involved. Here we found this switch between the different MAPK signals activity, ANLS rates, and HCA1 activity by various insults, indicating that lactate metabolic microenvironment plays a fundamental role in them (e.g., functional engagement of HCA1 receptor and its signaling). Totally, our data showed that both MCT2 and LDHB act in concert to regulate cerebral lactate homeostasis in aging mice in an HCA1‐independent manner.

Notably, in our previous studies with STZ‐induced T1D mice, reduced levels of MCT2 transporter led to less lactate flux to the cells and build‐up of the metabolite to higher levels in the extracellular space, subsequently activating HCA1 (Zhao, Dong, Ren, et al. 2018; Zhao, Dong, Wang, et al. 2018). Exogenous FGF21 treatment can increase neuronal lactate usage and learning and memory impairment in diabetic mice (Zhao et al. 2022). This extracellular‐specific action by HCA1 activation resembles the long‐term lactate direct treatment described in the study. This also confirmed that lactate did not accumulate in the extracellular space under an accelerated ANLS rate or aging conditions. Similarly, FGF21 treatment can also significantly increase ANLS rate in lactate mice via MCT2 overexpression. In the present study, elevated MCT2‐LDHB axis activity and ANLS rate in aging brains are attributed to endogenous FGF21 stimulation resulting from neuroinflammation‐induced insults. Interestingly, we also observed accelerated trends in PI3K‐mTOR activity and the MCT2‐LDHB axis in FGF21‐treated aging mice, suggesting that these signals respond to FGF21, which triggers PI3K signaling and facilitates the use of lactate in neurons.

The accelerated lactate metabolism in aging mice has not been studied, except for Dominika D.F. et al., who observed that aging neurons accelerate the synthesis of glycogen metabolism enzymes and glutamine synthase, accompanied by a rearrangement of the mitochondrial network (Drulis‐Fajdasz et al. 2018). Besides, we identified that decreased neuronal lactate uptake is sufficient to cause neuronal metabolic alterations and dysfunction. Here, for the first time, we propose that the rate of lactate uptake and usage (ANLS) may be related to neuronal activity, which leads to different learning and memory performance in mice. Due to the accelerated effects caused by FGF21 on ANLS rate, the elevation of FGF21 levels in normal aging brains might be a compensatory and protective action.

Although the liver is the major organ of FGF21 synthesis, adipocyte is its main target where FGF21 can increase glucose uptake and modulate lipolysis (Zhang et al. 2025; Li 2019). In the present study, western blot and IHC analysis confirmed a moderate hippocampal and cortical expression of the cytokine but lower than the liver tissues. Besides, we identified that accumulated FGF21 in aging brains mainly came from neurons, not other cells. Our data also suggested that FGF21 is produced in a P38‐dependent manner, and its site(s) of action were related to autocrine or paracrine. Analogously, it was reported that FGF21 expression was detected in the cortical and hippocampal neurons following mitochondrial dysfunction (Restelli et al. 2018). Sarah Geller et al. found that tanycytes, a subset of hypothalamic glial cells that sensed energy deprivation, produce and secrete FGF21 via a ROS/p38‐MAPK signaling pathway (Geller et al. 2019).

In aging brains, neurons not only use astrocyte‐derived lactate to boost bioenergetic levels but also secrete factors like FGF21 to amplify ANLS rates. We further illustrate that FGF21 sustains PI3K‐mTORC1 signal activation and MCT2 overexpression in neurons. These insights underscore the importance of modulating lactate metabolism in hippocampal and cortical neurons to enhance the efficacy of OXPHOS and ATP production, as well as protection from lactate accumulation. Our findings may expand our understanding of FGF21 in lactate metabolism beyond its known role in glucose and lipid homeostasis. Katsu‐Jiménez et al. observed that FGF21 can enhance the ability of cortical neurons to utilize ketone bodies by activating AMPK (Katsu‐Jimenez and Gimenez‐Cassina 2019). Indeed, it is well known that the Apolipoprotein E gene (APOE) is linked to a higher risk of AD (Goyal et al. 2023). Similarly, FGF21 variants, such as the rs838133 variant, have also been shown to serve as another high‐risk factor for AD (Wang et al. 2025; Larsson and Gill 2021).

## 5 Conclusions

In this work, we have examined the role of FGF21 in modulating the metabolic environment and lactate utilization in normal aging and lactate‐accumulating pathological circumstances. Altogether, our results suggest that cortical neuron‐secreted FGF21 may modulate cerebral lactate metabolism by priming neurons to obtain ATP from lactate more efficiently. There are many reports issued about the possible threshold of lactate homeostasis in the brain, or dual roles of lactate as a friend or foe (Wang et al. 2024; Taher et al. 2016; DiNuzzo 2016; Proia et al. 2016; Cai et al. 2022). For the first time, we proposed that the shuttle rate of lactate and the metabolic microenvironment are related to neuronal metabolism and function, but FGF21 can govern it. Moreover, due to the lactate metabolic disorder in AD, targeting lactate metabolism may be a novel way to treat AD.

There are several limitations that should be noted. First, Ariel K. Frame et al. reported that besides age, neuron‐generated lactate has sex‐specific effects on cognitive function in mice (Frame et al. 2024). However, in the present study, overlooking the potential contribution of female mice, we focus solely on male mice as the aging model. Secondly, in the present study, although FGF21 treatment for 4 weeks can effectively ameliorate learning and memory defects in aging and lactate mice, we do not know whether the 4‐week course is optimal for regulating L‐lactate homeostasis in vivo, and how long the effects of FGF21 could last after stopping its injection. Third, global KO models may develop compensatory changes in the mice; the HCA1 cell‐type‐specific KO mice need to be manipulated in our lab thereafter.

## Author Contributions

L.Z. and H.G. contributed to experimental design and writing of the manuscript. K.J., R.W., and Y.W. contributed to experiment data acquisition. K.J., H.W., and C.L. contributed to data analysis and result interpretation. All authors have read and approved the final manuscript.

## Funding

This study was supported by the Natural Science Foundation of Zhejiang Province (No. LY22H070003) and the National Natural Science Foundation of China (Nos. 22274115, 81770830).

## Conflicts of Interest

The authors declare no conflicts of interest.

## Supporting information


**Figure S1:** Increased neuroinflammation and lactate metabolism in naturally aging mice. (A) Immunofluorescence staining of p53 in the hippocampus and cortex regions of aging and control mice. (B) TUNEL staining of the hippocampus and cortex regions of the mice. (C) Representative western blots and densitometric quantifications of p‐AMPK, PI3K, P38, SOD2, C‐Fos, and Arc in the cortical extracts from the mice (*n* = 3). (D) Lactate levels in hippocampal extracts of the mice (*n* = 6). (E, F) Graphical RT‐PCR quantification of enzyme levels related to lactate metabolism and TCA cycle in hippocampal extracts of the mice (*n* = 6–7). (G) Colocalization of LDH‐B (green), NeuN (red), and merge (yellow) in the cortex and hippocampus regions of the aging mice. (H) Colocalization of LDH‐A (green), GFAP (red), and merge (yellow) in the cortex and hippocampus regions of the aging mice. Data are presented as mean ± SEM. *p < 0.05, **p < 0.01, ***p < 0.001, determined by student T‐test. n.s., not significant. “n” represents the number of mouse samples in each group.
**Figure S2:** Increased hippocampal FGF21 levels in naturally aging mice. (A) Graphical RT‐PCR quantification of FGF family members in hippocampal extracts of the mice (*n* = 6–7). (B) Representative western blots and densitometric quantifications of FGF21 in the hippocampal, cortical, and hepatic extracts of the mice (*n* = 4). (C) Immunofluorescence staining of FGF21 in the hippocampus, cortex, and hypothalamus regions of aging mice. (D) Colocalization of FGF21 (green), NeuN (marker: DCX), astrocytes (marker: GFAP), microglia (marker: IBA1), and merge (yellow) in the hippocampus DG regions of the aging mice. Data are presented as mean ± SEM. *p < 0.05, **p < 0.01, ***p < 0.001, determined by two‐tailed unpaired Student's *t* test. n.s., not significant. “n” represents the number of mouse samples in each group.
**Figure S3:** Representative western blots of p‐Nrf2, SLC7A11, and GPX4 in the cortical extracts from the mice (*n* = 3).
**Figure S4:** MCT2 attenuated both the ANLS pathway and learning and memory performance but not PI3K‐mTOR activity in aging mice. (A) A schematic diagram illustrates the strategy for stereotaxical adenovirus injections of sh‐MCT2 with GFP at the indicated locations in the mice. (B–E) MWM test: escape latency, path length, platform crossing, percentage of time spent in the TQ of mice with different treatments (*n* = 5–6). (F) Representative western blots and densitometric quantifications of mTOR‐PI3K and lactate metabolism pathways in the cortical extracts from the mice (*n* = 3). Data are presented as mean ± SEM. *p < 0.05, **p < 0.01, ***p < 0.001, determined by one‐way ANOVA followed by Turkey's multiple comparison test.
**Figure S5:** FGF21 administration ameliorated the learning and memory performance in mice and ANLS rate with long‐term lactate exposure. (A, B) Y‐maze: Path length and number crossing of mice with FGF21 and lactate treatment (*n* = 8–9). (C) LDH‐B activity in hippocampal extracts from the mice (*n* = 7). (D–G) Graphical RT‐PCR quantification of immediate early genes (IEGs) and synapse proteins in cortical extracts from the mice (*n* = 6). (H‐I) FGF21 dose and time‐dependently increased ANLS‐related protein and p‐AMPK levels in SH‐SY5Y cells. Data are presented as mean ± SEM. *p < 0.05, **p < 0.01, ***p < 0.001, determined by two‐way ANOVA and followed by Dunnett multiple comparison test. n.s., not significant. AL: Adjacent left; OP: Opposite; TQ: Target quadrant; AR: Adjacent right.
**Figure S6:** Lactate flux assay of primary neurons and astrocytes, respectively. (A) Cell culture schematics were performed to assess the cellular lactate flux process. (B) Enzymatic assay quantifying lactate concentration in the media of neurons treated with lactate or vehicle under the indicated concentrations according to A (*n* = 3). (C, D) Enzymatic assay quantifying lactate concentration in the media of neurons treated with L‐lactate, FGF21, or MCT inhibitors AR‐C155858, or sh‐MCT2 (*n* = 3). (E‐F) Enzymatic assay quantifying lactate concentration in the media of neurons (E) and astrocytes (F) treated with glucose (25 mM) or MCT inhibitors (α‐CHC, AR‐C155858) for the indicated time points (*n* = 3). Data are presented as mean ± SEM. *p < 0.05, **p < 0.01, ***p < 0.001, determined by one‐way ANOVA and followed by Turkey's multiple comparison test. n.s., not significant.
**Figure S7:** Long‐term lactate treatment attenuated PKA‐CREB activity in neurons via an HCA1‐dependent manner. (A) Lactate treated primary neurons for 36 h significantly inhibited pCREB expression, which could be mimicked by HCA receptor agonist 3,5‐DHBA (*n* = 3). (B) Inhibited effects of lactate treatment on pCREB expression could be prevented by concomitant of adenylate cyclase (AC) agonist forskolin (*n* = 3). (C) Inhibited effects of lactate treatment on pCREB and pPKA expression could be found in HEK293T cells with HCA1 overexpression (*n* = 3). (D) Inhibited cortical pCREB and pPKA expression could be found in lactate‐infused mice but not aging mice (*n* = 3). Data are presented as mean ± SEM. *p < 0.05, **p < 0.01, ***p < 0.001, determined by one‐way ANOVA and followed by Turkey's multiple comparison test. n.s., not significant.
**Figure S8:** HCA1 is not involved in cortical ANLS acceleration and FGF21 production in aging mice. (A) A schematic diagram illustrates the strategy for knocking out HCA1. (B‐C) Agarose gel validates HCA1‐KO efficacy. DNA fragments from HCA1^+/+^ (541 bp), HCA1^−/−^ (704 bp), and marker DNA are indicated. (D, E) Y‐maze: Path length and crossing number of the mice (*n* = 5). (F) Representative western blots and densitometric quantifications of p‐AMPK, MCT2, LDH‐B, c‐Fos, and FGF21 in the cortical extracts from the mice (*n* = 3). Data are presented as mean ± SEM. *p < 0.05, **p < 0.01, ***p < 0.001, two‐way ANOVA and followed by Dunnett multiple comparison test.
**Figure S9:** H_2_O_2_ treatment induced senescence and ROS levels in SH‐SY5Y cells. (A) Experimental conditions: untreated cells serve as the control group (control), with the model group (H_2_O_2_ concentrations: 400 μM). (B) SA‐β‐galactosidase staining was measured at 24 h after H_2_O_2_ treatment. (C) Representative western blots and densitometric quantifications of P53, P21, P16, NOX, SOD, Bax, and Bcl‐2 in the extracts of SH‐SY5Y cells (*n* = 4). (D) Representative images of dihydroethidium (DHE) staining in the cells and quantitative analysis of fluorescent intensity. Data are presented as mean ± SEM. *p < 0.05, **p < 0.01, ***p < 0.001, determined by two‐tailed unpaired Student's *t* test. n.s., not significant. “*n*” represents the number of mouse samples in each group.
**Figure S10:** P38‐mediated FGF21 production and PI3K‐mTOR‐dependent MCT2 protein are involved in the effects of FGF21 on neurons. (A) Representative western blots and densitometric quantifications of JNK, ERK1/2, and p38 MAPK phosphorylation, and FGF21 levels in the primary neurons treated by H_2_O_2_ for 24 h (*n* = 4). (B) Representative images of immunofluorescence staining of FGF21 in the cells and quantitative analysis of fluorescent intensity (*n* = 4). (C) Primary neurons were treated with H_2_O_2_ or PBS for 24 h in the presence of ERK1/2 inhibitor (PD98059, 20 μM), JNK inhibitor (SP600125, 20 μM), or p38 inhibitor (SB1290, 10 μM). Then, immunofluorescence staining of FGF21 in the neurons was examined. (D) Primary neurons were treated with FGF21 or PBS for 24 h in the presence of mTOR inhibitor (Rapamycin, 20 nM) and PI3K inhibitor (LY294002, 20 μM). Then, immunofluorescence staining of MCT2 in the neurons was examined. (E) Schematic diagram of p38‐mediated FGF21 production and PI3K‐mTOR pathway‐mediated MCT2 expression involved in the effects of FGF21 on neurons. Data are presented as mean ± SEM. *p < 0.05, **p < 0.01, ***p < 0.001, determined by two‐tailed unpaired Student's *t* test (a, b) and two‐way ANOVA and followed by Dunnett multiple comparison test (c, d).

## Data Availability

The data that support the findings of this study are available on request from the corresponding author. The data are not publicly available due to privacy or ethical restrictions.
