# Supplementary material for: Acceleration of Lactate Uptake and Utilization Contributes to Neuroprotective Action of FGF21 Involved in Naturally Aging Mice
Source: Aging Cell. 2026 Feb 21;25(3):e70423. doi: 10.1111/acel.70423 (PMC12928016; doi:10.1111/acel.70423)
Supplement: Supplementary file 1 — Figure S1: Increased neuroinflammation and lactate metabolism in naturally aging mice. (A) Immunofluorescence staining of p53 in the hippocampus and cortex regions of aging and control mice. (B) TUNEL staining of the hippocampus and cortex regions of the mice. (C) Representative western blots and densitometric quantifications of p‐AMPK, PI3K, P38, SOD2, C‐Fos, and Arc in the cortical extracts from the mice (n = 3). (D) Lactate levels in hippocampal extracts of the mice (n = 6). (E, F) Graphical RT‐PCR quantification of enzyme levels related to lactate metabolism and TCA cycle in hippocampal extracts of the mice (n = 6–7). (G) Colocalization of LDH‐B (green), NeuN (red), and merge (yellow) in the cortex and hippocampus regions of the aging mice. (H) Colocalization of LDH‐A (green), GFAP (red), and merge (yellow) in the cortex and hippocampus regions of the aging mice. Data are presented as mean ± SEM. *p < 0.05, **p < 0.01, ***p < 0.001, determined by student T‐test. n.s., not significant. “n” represents the number of mouse samples in each group. Figure S2: Increased hippocampal FGF21 levels in naturally aging mice. (A) Graphical RT‐PCR quantification of FGF family members in hippocampal extracts of the mice (n = 6–7). (B) Representative western blots and densitometric quantifications of FGF21 in the hippocampal, cortical, and hepatic extracts of the mice (n = 4). (C) Immunofluorescence staining of FGF21 in the hippocampus, cortex, and hypothalamus regions of aging mice. (D) Colocalization of FGF21 (green), NeuN (marker: DCX), astrocytes (marker: GFAP), microglia (marker: IBA1), and merge (yellow) in the hippocampus DG regions of the aging mice. Data are presented as mean ± SEM. *p < 0.05, **p < 0.01, ***p < 0.001, determined by two‐tailed unpaired Student's t test. n.s., not significant. “n” represents the number of mouse samples in each group. Figure S3: Representative western blots of p‐Nrf2, SLC7A11, and GPX4 in the cortical extracts from the mice (n = 3). Figure [file ACEL-25-e70423-s001.docx]

**Supplementary materials:**


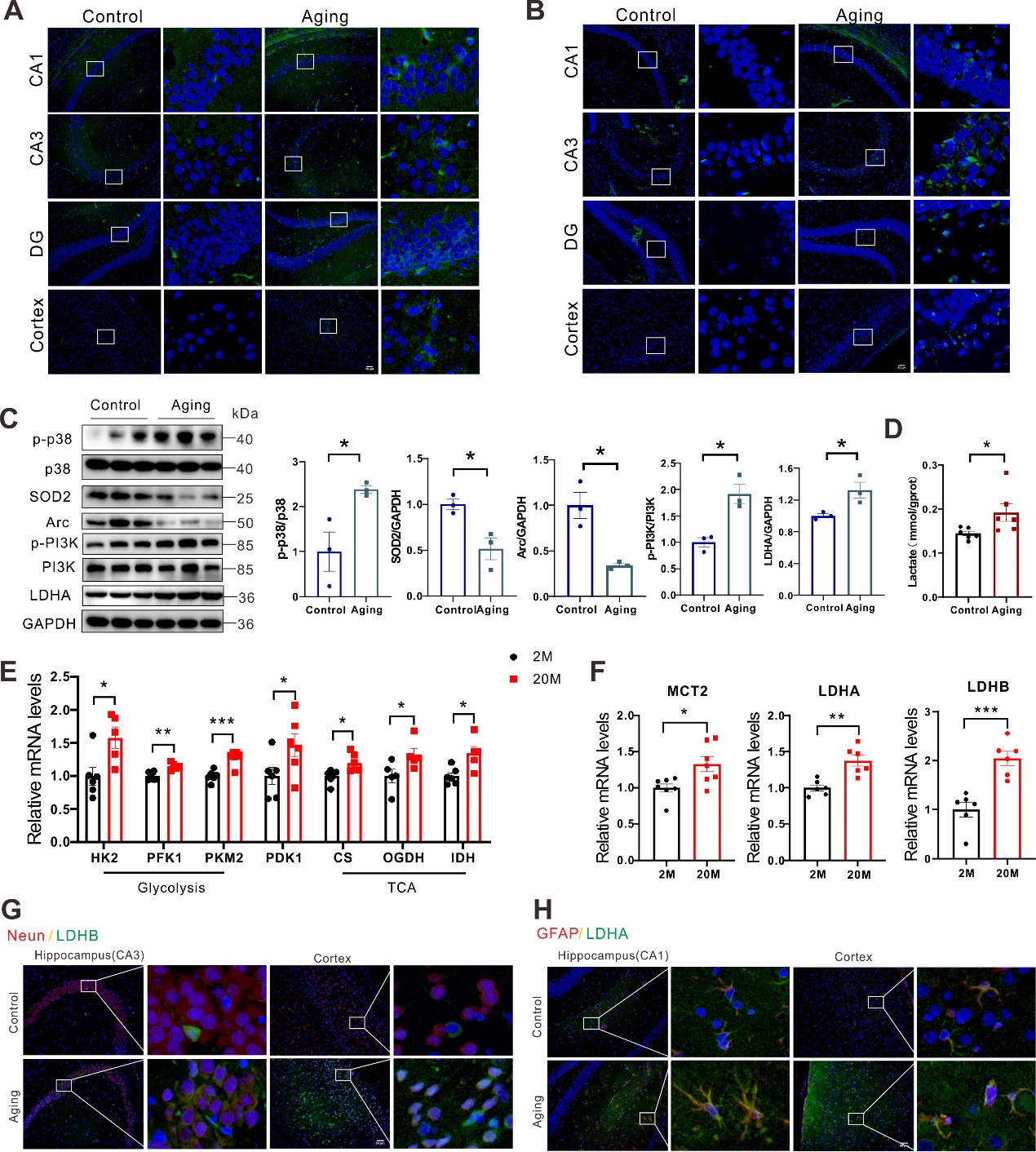


**Fig. S1 Increased neuroinflammation and lactate metabolism in naturally aging mice.** (A) Immunofluorescence staining of p53 in the hippocampus and cortex regions of aging and control mice. (B) TUNEL staining of the hippocampus and cortex regions of the mice. (C) Representative western blots and densitometric quantifications of p-AMPK, PI3K, P38, SOD2, C-Fos, and Arc in the cortical extracts from the mice (n = 3). (D) Lactate levels in hippocampal extracts of the mice (n = 6). (E-F) Graphical RT-PCR quantification of enzyme levels related to lactate metabolism and TCA cycle in hippocampal extracts of the mice (n = 6-7). (G) Colocalization of LDH-B (green), NeuN (red), and merge (yellow) in the cortex and hippocampus regions of the aging mice. (H) Colocalization of LDH-A (green), GFAP (red), and merge (yellow) in the cortex and hippocampus regions of the aging mice. Data are presented as mean ± SEM. *p < 0.05, **p < 0.01, ^***^p < 0.001, determined by student T-test. n.s., not significant. “n” represents the number of mouse samples in each group.


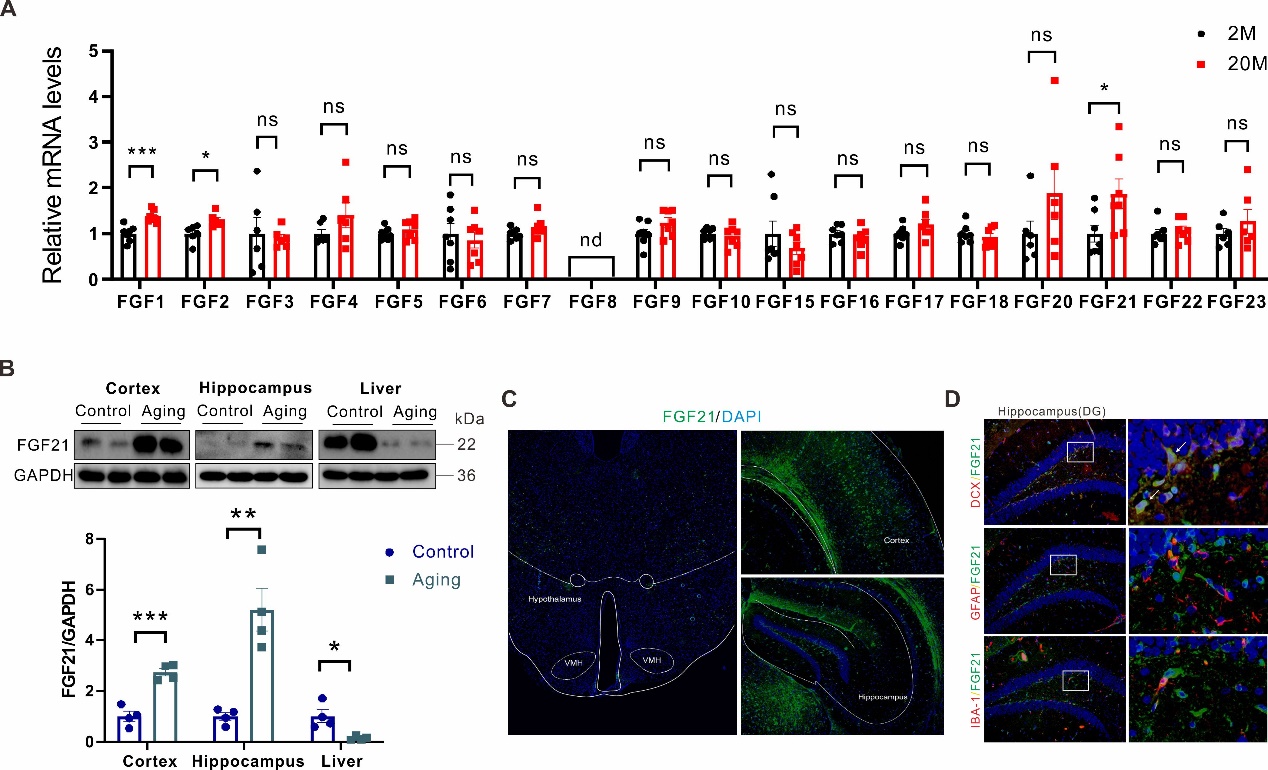


**Fig. S2 Increased hippocampal FGF21 levels in naturally aging mice.** (A) Graphical RT-PCR quantification of FGF family members in hippocampal extracts of the mice (n = 6-7). (B) Representative western blots and densitometric quantifications of FGF21 in the hippocampal, cortical, and hepatic extracts of the mice (n = 4). (C) Immunofluorescence staining of FGF21 in the hippocampus, cortex, and hypothalamus regions of aging mice. (D) Colocalization of FGF21 (green), NeuN (marker: DCX), astrocytes (marker: GFAP), microglia (marker: IBA1), and merge (yellow) in the hippocampus DG regions of the aging mice. Data are presented as mean ± SEM. *p < 0.05, **p < 0.01, ^***^p < 0.001, determined by two-tailed unpaired Student’s t test. n.s., not significant. “n” represents the number of mouse samples in each group.


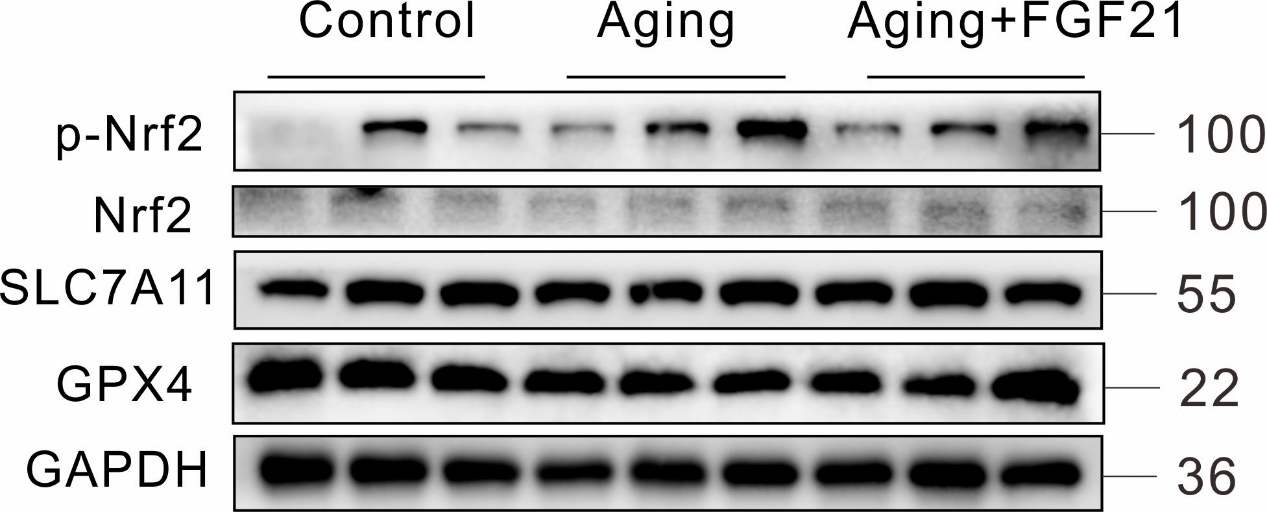


**Fig. S3 Representative western blots of p-Nrf2, SLC7A11, and GPX4 in the cortical extracts from the mice (n = 3).**


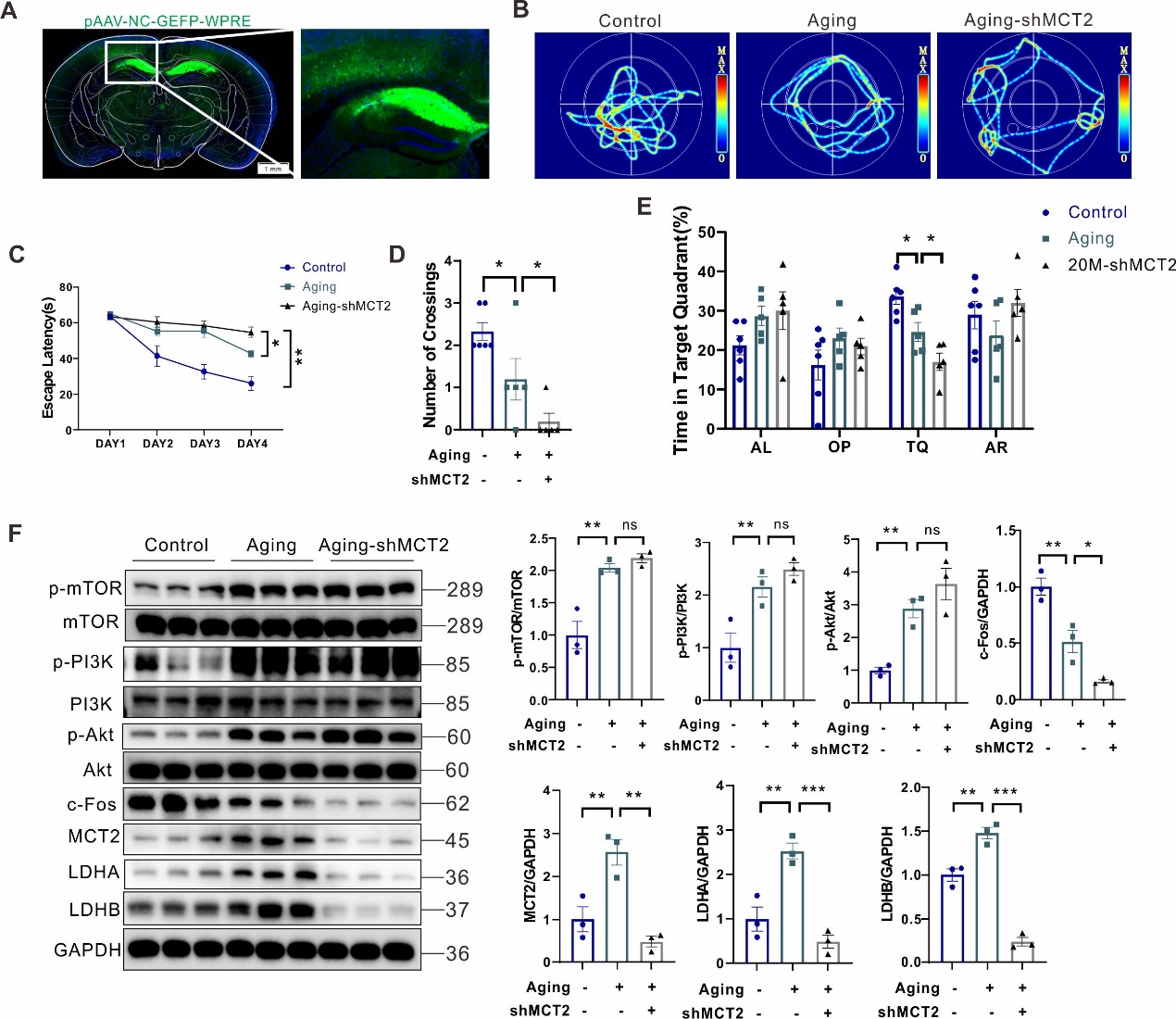


**Fig. S4 MCT2 attenuated both the ANLS pathway and learning and memory performance but not PI3K-mTOR activity in aging mice.** (A) A schematic diagram illustrates the strategy for stereotaxical adenovirus injections of sh-MCT2 with GFP at the indicated locations in the mice. (B-E) MWM test: escape latency, path length, platform crossing, percentage of time spent in the TQ of mice with different treatments (n = 5-6). (F) Representative western blots and densitometric quantifications of mTOR-PI3K and lactate metabolism pathways in the cortical extracts from the mice (n = 3). Data are presented as mean ± SEM. *p < 0.05, **p < 0.01, ^***^p < 0.001, determined by one-way ANOVA followed by Turkey’s multiple comparison test.


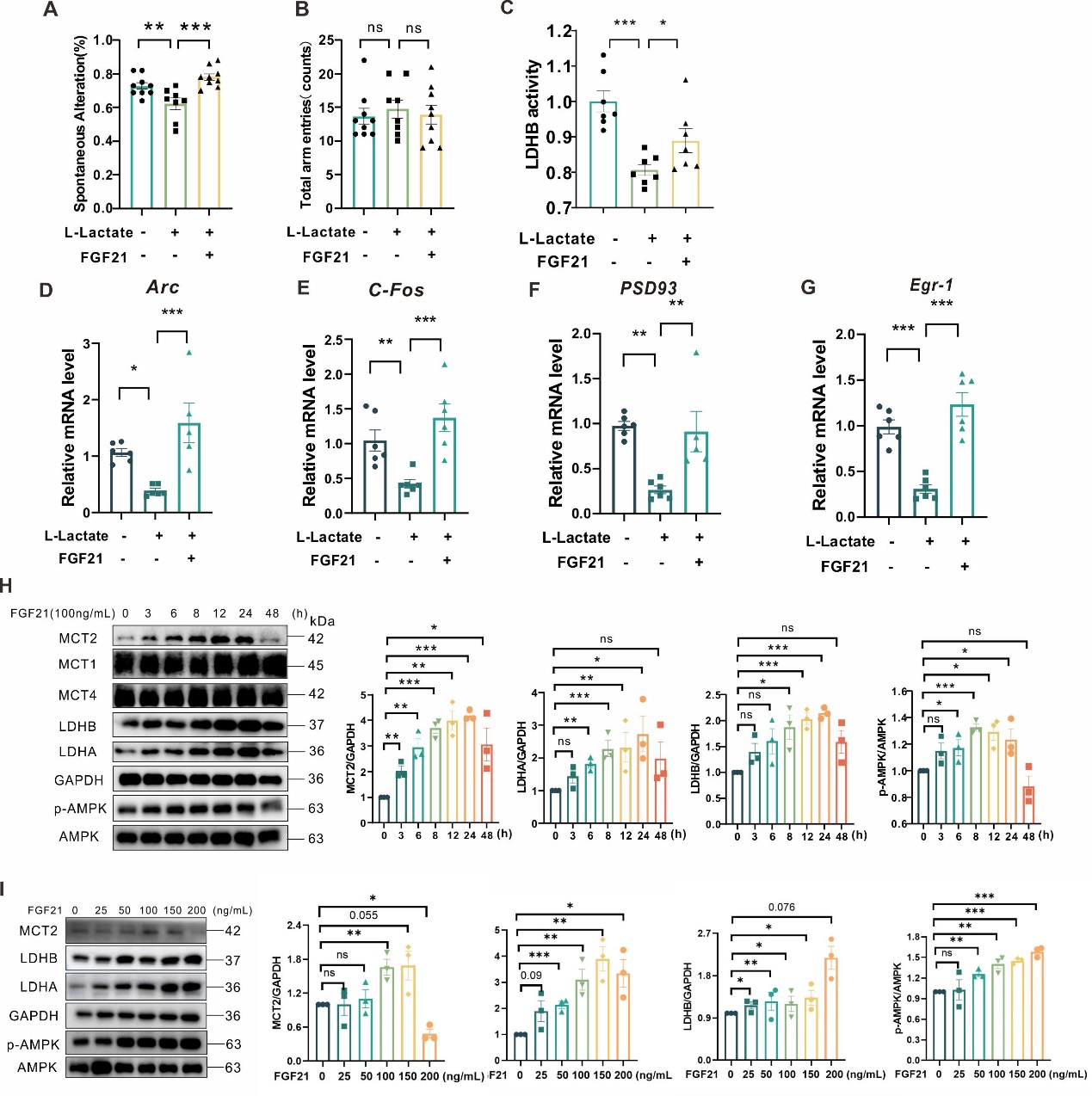


**Fig. S5 FGF21 administration ameliorated the learning and memory performance in mice and ANLS rate with long-term lactate exposure.** (A-B) Y-maze: Path length and number crossing of mice with FGF21 and lactate treatment (n = 8-9). (C) LDH-B activity in hippocampal extracts from the mice (n = 7). (D-G) Graphical RT-PCR quantification of immediate early genes (IEGs) and synapse proteins in cortical extracts from the mice (n = 6). (H-I) FGF21 dose and time-dependently increased ANLS-related protein and p-AMPK levels in SH-SY5Y cells. Data are presented as mean ± SEM. *p < 0.05, **p < 0.01, ***p < 0.001, determined by two-way ANOVA and followed by Dunnett multiple comparison test. n.s., not significant. AL: Adjacent left; OP: Opposite; TQ: Target quadrant; AR: Adjacent right.


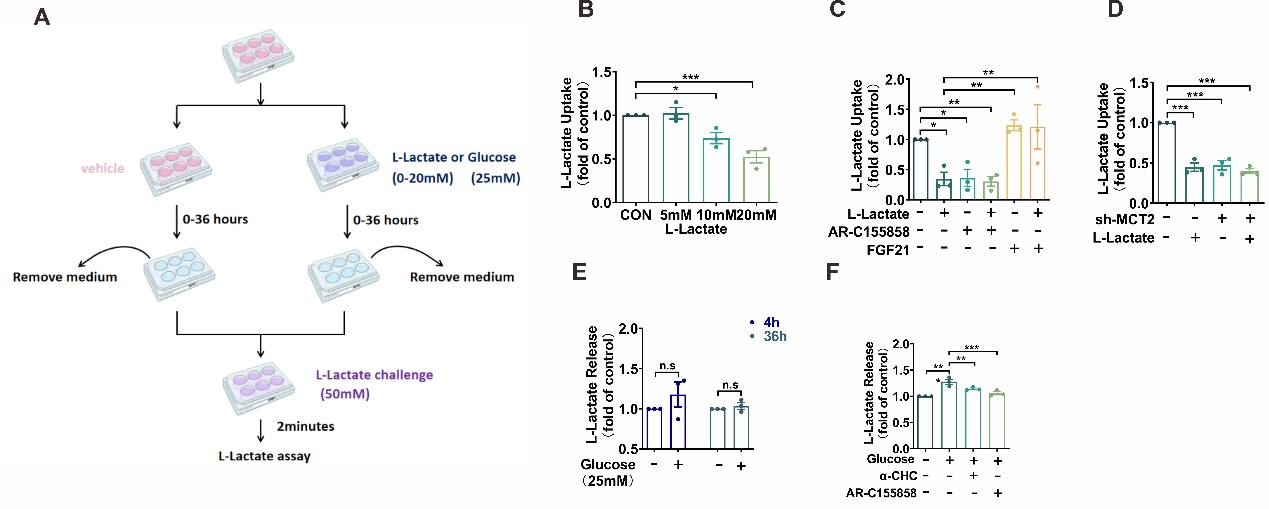


**Fig. S6 Lactate flux assay of primary neurons and astrocytes, respectively.** (A) Cell culture schematics were performed to assess the cellular lactate flux process. (B) Enzymatic assay quantifying lactate concentration in the media of neurons treated with lactate or vehicle under the indicated concentrations according to A (n = 3). (C-D) Enzymatic assay quantifying lactate concentration in the media of neurons treated with L-lactate, FGF21, or MCT inhibitors AR-C155858, or sh-MCT2 (n = 3). (E-F) Enzymatic assay quantifying lactate concentration in the media of neurons (E) and astrocytes (F) treated with glucose (25 mM) or MCT inhibitors (α-CHC, AR-C155858) for the indicated time points (n = 3). Data are presented as mean ± SEM. *p < 0.05, **p < 0.01, ***p < 0.001, determined by one-way ANOVA and followed by Turkey’s multiple comparison test. n.s., not significant.


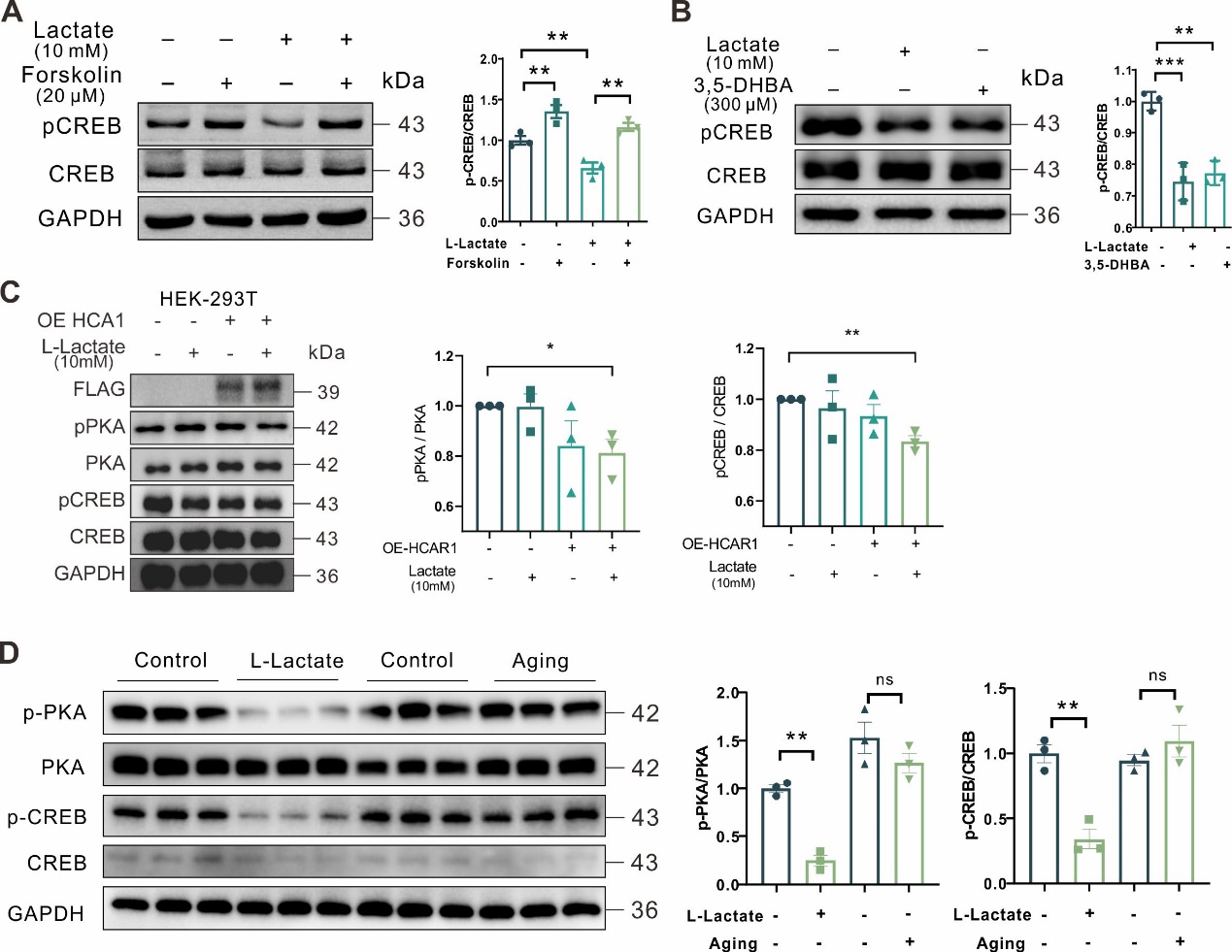


**Fig. S7 Long-term lactate treatment attenuated PKA-CREB activity in neurons via an HCA1-dependent manner.** (A) Lactate treated primary neurons for 36 hours significantly inhibited pCREB expression, which could be mimicked by HCA receptor agonist 3,5-DHBA (n = 3). (B) Inhibited effects of lactate treatment on pCREB expression could be prevented by concomitant of adenylate cyclase (AC) agonist forskolin (n = 3). (C) Inhibited effects of lactate treatment on pCREB and pPKA expression could be found in HEK293T cells with HCA1 overexpression (n = 3). (D) Inhibited cortical pCREB and pPKA expression could be found in lactate-infused mice but not aging mice (n = 3). Data are presented as mean ± SEM. *p < 0.05, **p < 0.01, ***p < 0.001, determined by one-way ANOVA and followed by Turkey’s multiple comparison test. n.s., not significant.


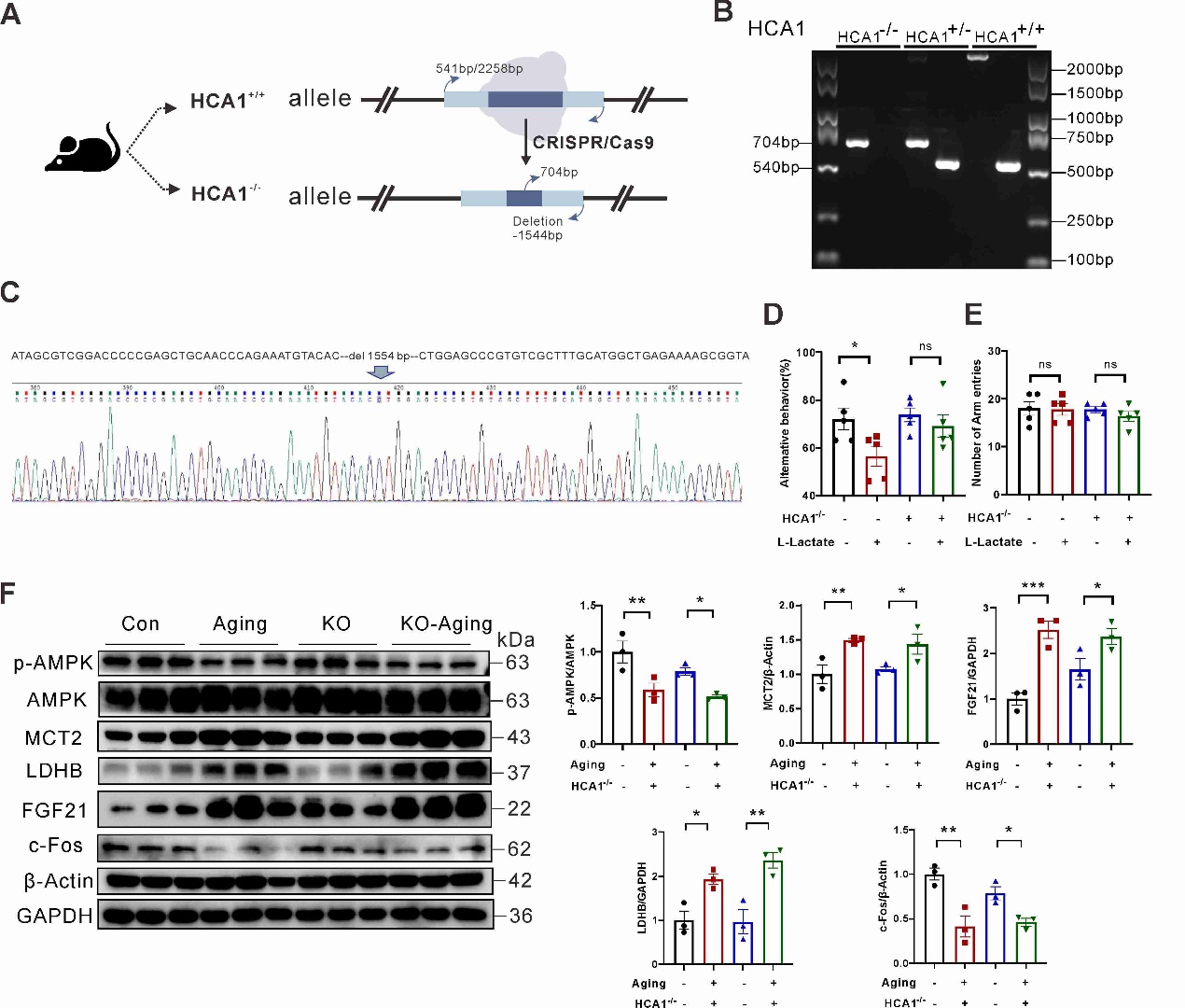


**Fig. S8 HCA1 is not involved in cortical ANLS acceleration and FGF21 production in aging mice.** (A) A schematic diagram illustrates the strategy for knocking out HCA1. (B-C) Agarose gel validates HCA1-KO efficacy. DNA fragments from HCA1^+/+^ (541 bp), HCA1^-/-^ (704 bp), and marker DNA are indicated. (D-E) Y-maze: Path length and crossing number of the mice (n = 5). (F) Representative western blots and densitometric quantifications of p-AMPK, MCT2, LDH-B, c-Fos, and FGF21 in the cortical extracts from the mice (n = 3). Data are presented as mean ± SEM. *p < 0.05, **p < 0.01, ***p < 0.001, two-way ANOVA and followed by Dunnett multiple comparison test.


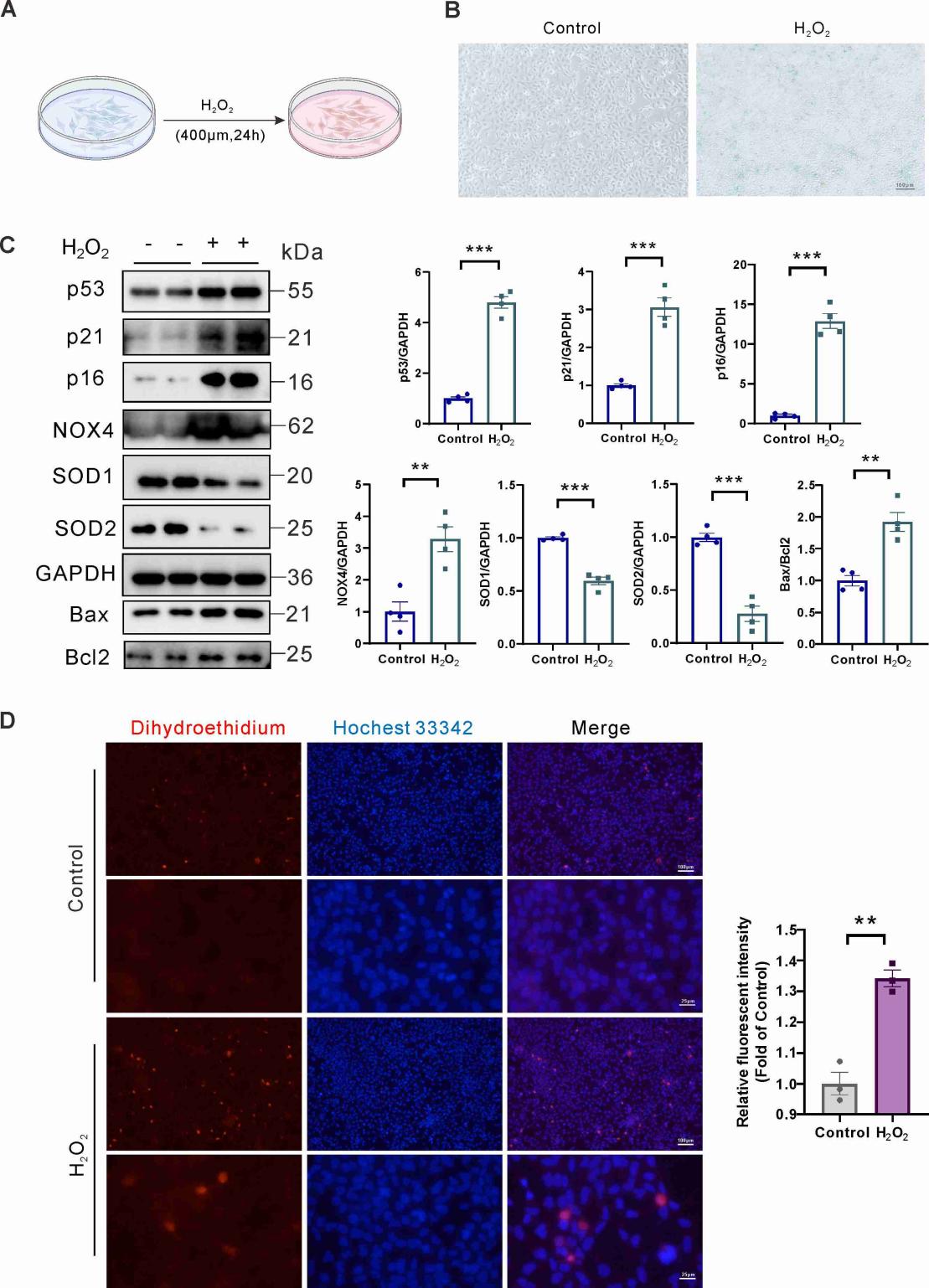


**Fig. S9** H_2_O_2_ treatment induced senescence and ROS levels in SH-SY5Y cells. (A) Experimental conditions: untreated cells serve as the control group (control), with the model group (H_2_O_2_ concentrations: 400 μM). (B) SA-β-galactosidase staining was measured at 24 h after H_2_O_2_ treatment. (C) Representative western blots and densitometric quantifications of P53, P21, P16, NOX, SOD, Bax, and Bcl-2 in the extracts of SH-SY5Y cells (n = 4). (D) Representative images of dihydroethidium (DHE) staining in the cells and quantitative analysis of fluorescent intensity. Data are presented as mean ± SEM. *p < 0.05, **p < 0.01, ^***^p < 0.001, determined by two-tailed unpaired Student’s t test. n.s., not significant. “n” represents the number of mouse samples in each group.


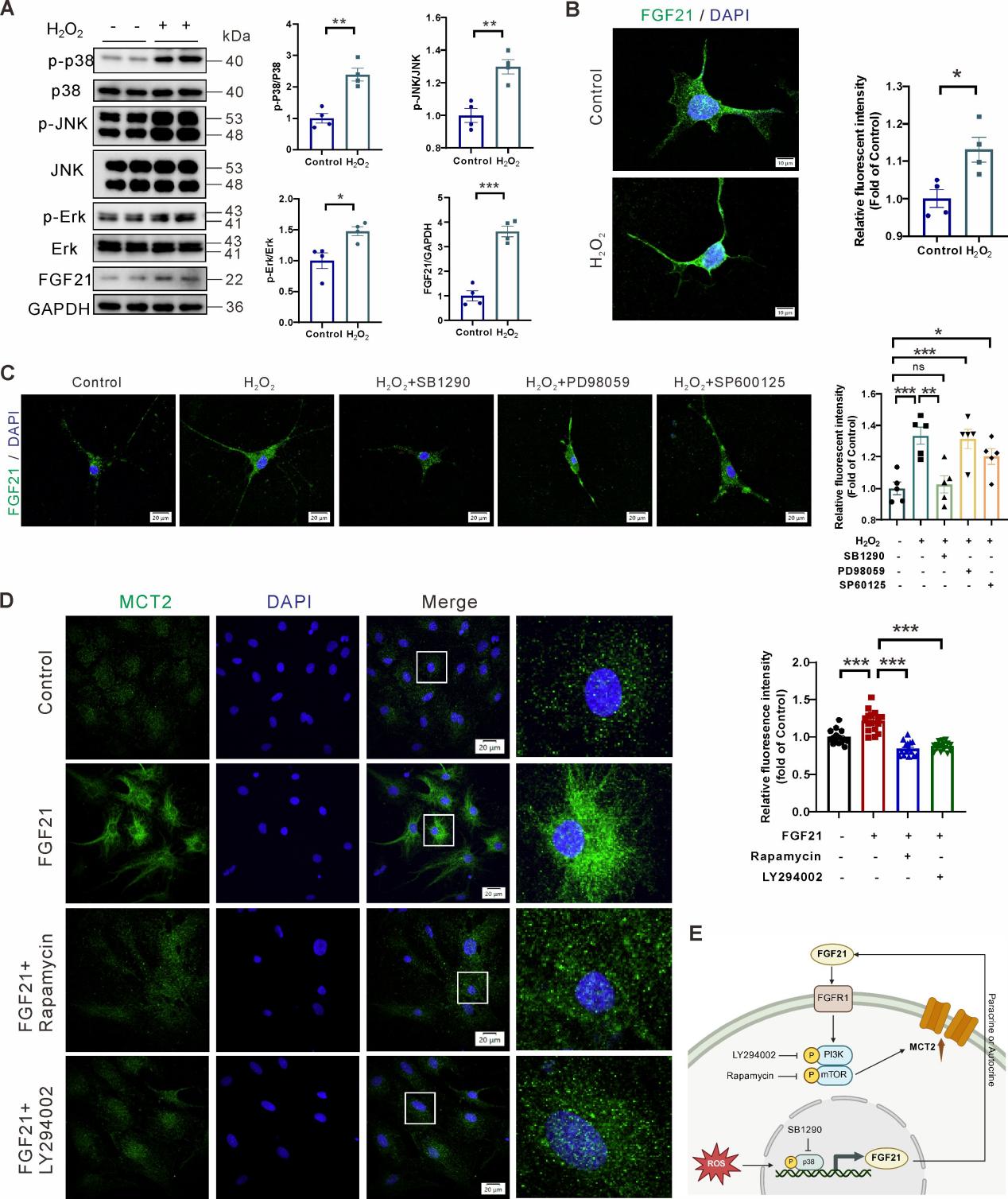


**Fig. S10 P38-mediated FGF21 production and PI3K-mTOR-dependent MCT2 protein are involved in the effects of FGF21 on neurons.** (A) Representative western blots and densitometric quantifications of JNK, ERK1/2, and p38 MAPK phosphorylation, and FGF21 levels in the primary neurons treated by H_2_O_2_ for 24 h (n = 4). (B) Representative images of immunofluorescence staining of FGF21 in the cells and quantitative analysis of fluorescent intensity (n = 4). (C) Primary neurons were treated with H_2_O_2_ or PBS for 24 h in the presence of ERK1/2 inhibitor (PD98059, 20 μM), JNK inhibitor (SP600125, 20 μM), or p38 inhibitor (SB1290, 10 μM). Then, immunofluorescence staining of FGF21 in the neurons was examined. (D) Primary neurons were treated with FGF21 or PBS for 24 h in the presence of mTOR inhibitor (Rapamycin, 20 nM) and PI3K inhibitor (LY294002, 20 μM). Then, immunofluorescence staining of MCT2 in the neurons was examined. (E) Schematic diagram of p38-mediated FGF21 production and PI3K-mTOR pathway-mediated MCT2 expression involved in the effects of FGF21 on neurons. Data are presented as mean ± SEM. ^*^p < 0.05, ^**^p < 0.01, ^***^p < 0.001, determined by two-tailed unpaired Student’s t test (a, b) and two-way ANOVA and followed by Dunnett multiple comparison test (c, d).
